# An accurate, robust, and efficient finite element framework with applications to anisotropic, nearly and fully incompressible elasticity

**DOI:** 10.1016/j.cma.2022.114887

**Published:** 2022-03-31

**Authors:** Elias Karabelas, Matthias A.F. Gsell, Gundolf Haase, Gernot Plank, Christoph M. Augustin

**Affiliations:** a Institute for Mathematics and Scientific Computing, Karl-Franzens-University Graz, Graz, Austria; b Gottfried Schatz Research Center: Division of Biophysics, Medical University of Graz, Graz, Austria; c BioTechMed-Graz, Graz, Austria

**Keywords:** Stabilized finite element methods, Anisotropic materials, Quasi-incompressibility, Soft biological tissues, Cardiac electromechanics

## Abstract

Fiber-reinforced soft biological tissues are typically modeled as hyperelastic, anisotropic, and nearly incompressible materials. To enforce incompressibility a multiplicative split of the deformation gradient into a volumetric and an isochoric part is a very common approach. However, the finite element analysis of such problems often suffers from severe volumetric locking effects and numerical instabilities. In this paper, we present novel methods to overcome volumetric locking phenomena for using stabilized P1–P1 elements. We introduce different stabilization techniques and demonstrate the high robustness and computational efficiency of the chosen methods. In two benchmark problems from the literature as well as an advanced application to cardiac electromechanics, we compare the approach to standard linear elements and show the accuracy and versatility of the methods to simulate anisotropic, nearly and fully incompressible materials. We demonstrate the potential of this numerical framework to accelerate accurate simulations of biological tissues to the extent of enabling patient-specific parameterization studies, where numerous forward simulations are required.

## 1 Introduction

Computer models of biological tissues, e.g., the simulation of vessel mechanics or cardiac electro-mechanics (EM), aid in understanding the biomechanical function of the organs and show promise to be a powerful tool for predicting therapeutic responses. Advanced applications include simulations to assess passive filling properties and active response to pacing therapies [1], simulations of growth and remodeling processes occurring in the failing heart or arteries [2–5], as well as rupture risk assessment in arterial aneurysms [6,7]. Here, predictions of *in-silico* models are often based on the computation of local stresses, hence, an accurate computation of strain and stress is indispensable to achieve mechanistically sound predictions. To build confidence in simulation outcomes proper model calibration, validation, uncertainty quantification, and sensitivity analyses are required. Additionally, high computational efficiency and excellent strong scaling properties are crucial to perform simulations with highly-resolved, complex, or heterogeneous geometries within tractable time frames; to facilitate model personalization using a large number of forward simulations; and to simulate tissue behavior over a broad range of experimental protocols and extended observation periods.

*In-silico* models of cardiac tissue and vessel walls are typically based on the theory of hyperelasticity and properties of soft tissues include a nonlinear relationship between stress and strain with large deformations and a nearly-incompressible, anisotropic materials [8–10]. Commonly, the resulting non-linear formulations are approximately solved using a finite element (FE) approach [11–14]. However, volumetric locking phenomena, that are resulting in ill-conditioned global stiffness matrices are frequently encountered. Here and in the following, we use the definition of locking given in the seminal work of Babuška and Suri [15], meaning the dependance of convergence of a FE solution on a critical parameter – in our applications the bulk modulus *κ* – and the lack thereof in the limit. In fact, this is one of the classical problems of modeling nearly incompressible hyperelasticity [16,17]. Volumetric locking, often completely invalidating FE solutions, is in particular prevalent for fiber-reinforced soft biological tissues due to a high stiffness in the preferred fiber directions compared to a relatively soft matrix material [18] and is thus extensively studied in recent publications [19–22].

Typically, the modeling of (nearly) incompressible elastic materials involves a split of the deformation gradient into a volumetric and an isochoric part [23]. Here, volumetric locking phenomena are very common when using unstable approximation pairs such as Q1–P0 or P1–P0 elements, i.e., when linear shape functions are the choice to approximate the displacement field ***u*** and the hydrostatic pressure *p* is statically condensed from the system of equations on the element level. It is well known that in such cases solution algorithms are likely to show very low convergence rates, and that variables of interest such as stresses can be inaccurate [20].

To some degree volumetric locking problems in anisotropic hyperelasticity for these simple elements can be reduced by using augmented Lagrangian methods [24,25], formulations with an unsplit deformation gradient for the anisotropic contribution [26,27], and methods with simplified kinematics for the anisotropic contributions [22]. Another possibility to obtain more accurate results is the use of higher order polynomials to approximate the displacement [28–31]. However, the incompressibility constraint is still modeled by a penalty formulation, hence, volumetric locking may still be an issue and the modeling of fully incompressible materials is not possible. Additionally, already for quadratic ansatz functions the considerable larger amount of degrees of freedom increases computational cost significantly.

A more sophisticated approach – also allowing the modeling of fully incompressible materials – is the reformulation of the underlying equations into a saddle point problem by introducing the hydrostatic pressure *p* as an additional unknown to the system. Here, from mathematical theory, approximation pairs for ***u*** and *p* have to fulfill the Ladyzhenskaya–Babuŝka–Brezzi (LBB) or *inf–sup* conditions [32–34] to guarantee stability. A popular choice are quadratic ansatz functions for the displacement and linear ansatz functions for the pressure, i.e., the Taylor–Hood element [35,36]. Though stable, this element leads to a vast increase in degrees of freedom and the development of scalable solvers is very difficult. Consequently, this entails a high computational burden; especially for applied problems in the field of tissue mechanics with highly resolved geometries.

A computationally potentially more favorable choice are equal order pairs with a stabilization, widely used for linear and isotropic elasticity [37–43]. Yet, their extension to non-linear problems is challenging [22,44,45]. In the specific case of modeling biological tissues Hu–Washizu-based formulations are often used, e.g., [46–49]. However, especially for problems undergoing large strains, this mixed three field approach shows limited performance and robustness [22]. Further, variational multiscale (VMS) formulations have been used for nonlinear solid mechanics [39,50,51]. VMS is frequently used in computational fluid mechanics with the key advantage that these formulations offer both stabilization as well as benefits in turbulence modeling. The main idea behind VMS is to split FE solutions into resolvable and unresolvable parts, where the unresolved parts of the solution are modeled with the residual of the physics. On the other hand, VMS formulations are introduced at the discrete FE level, and thus one usually obtains modified variational formulations that include mesh-depended parameters. For solid mechanics, introducing VMS results in a modified FE formulation that can encode anisotropic material behavior very well [39]. However, this comes at the cost of a very intrusive change into the formulation as the consistent linearization introduces non-standard 6th order tensors which need to be implemented into existing code frameworks.

A very promising and efficient stabilization approach for nearly incompressible elasticity problems is a variant of the MINI element [52], originally established for computational fluid dynamics problems. This element is modified for the application of incompressible hyperelasticity and a bubble function is included in the ansatz space of displacements. To improve efficiency, the support of this bubble can be eliminated from the system of equation using static condensation. First uses of MINI elements have been reported [53,54] though still using a piecewise constant ansatz for the hydrostatic pressure. Even more efficient and notably simpler to implement is a pressure projection method originally introduced for the Stokes problem [55]. To the best of our knowledge the here proposed methods were not yet applied in this form to anisotropic and nearly incompressible materials.

A significant advantage of both stabilization techniques is that these do not rely on artificial stabilization parameters that may influence the numerical solution. We illustrate in different benchmarks that the same setting can be used for a large variety of tissue mechanics problems allowing for a one-for-all approach. By comparing to other methods previously reported in the literature, we show that our methods are suitable to compute accurate strain and stress fields and outperform existing contributions in terms of efficiency.

The paper is outlined as follows: in Section 2 we recall the mathematical background of modeling anisotropic, nearly incompressible elasticity and introduce the theoretical framework of our stabilization techniques. Subsequently, Section 3 documents three benchmarks problems to show the applicability of the stabilized P1-P1 elements in different scenarios. For each benchmark we give a detailed problem description and discuss results and computational efficiency by comparing to the literature and analyzing strong-scaling properties.

To show the usefulness of the presented methods to clinically relevant problems, we present a 3D EM model of the heart that is coupled to a 0D model of the circulatory system. This constitutes one of the most complete model of cardiac EM in the literature to date as all components, i.e., electrophysiology, cellular dynamics, active stress, passive tissue mechanics, pre- and afterload, are based on physiological, state-of-the-art models. Finally, Section 4 concludes the paper with a brief summary and all required equations to implement the methods in a software framework are given in Appendix A.

## 2 Methodology

### 2.1 Almost incompressible nonlinear elasticity

Let *Ω*_0_ ⊂ ℝ^3^ denote the reference configuration and let *Ω_t_* ⊂ ℝ^3^ denote the current configuration of the domain of interest. Assume that the boundary of *Ω*_0_ is decomposed into *∂Ω*_0_ = *Γ*_D_,_0_ ∪ *Γ*_N_,_0_ with | *Γ*_D_,_0_| > 0. Here, *Γ*_D_,_0_ describes the Dirichlet part of the boundary and *Γ*_N,0_ describes the Neumann part of the boundary, respectively. Further, let ***N*** be the unit outward normal on *∂Ω*_0_. The nonlinear mapping ***ϕ*** : ***X*** ∈ *Ω*_0_ → ***x*** ∈ *Ω_t_*), defined by ***ϕ*** ≔ ***X*** + ***u***(***X***, *t*), with displacement ***u***, maps points in the reference configuration to points in the current configuration. Following standard notation, we introduce the *deformation gradient **F***, the Jacobian *J*, and the *left Cauchy–Green tensor **C*** as

$$\begin{array}{ccc} \text{F}≔\text{Grad}\;\phi =\text{I}+\text{Grad}\;\text{u}, & J≔\text{det}(\text{F}), & \text{C}≔{\text{F}}^{⊤}\text{F}. \end{array}$$

Here, Grad(•) denotes the gradient with respect to the reference coordinates ***X*** ∈ *Ω*_0_. The displacement field ***u*** is sought as infimizer of the functional

(1) $$\begin{array}{lll} \Pi^{\text{tot}}(\text{u}) & ≔ & \Pi (\text{u})+\Pi^{\text{ext}}(\text{u}),\\ \Pi (\text{u}) & ≔ & \int_{\Omega_{0}}\Psi (\text{F}(\text{u}))\text{d}\text{X},\\ \Pi^{\text{ext}}(\text{u}) & ≔ & -\rho_{0}\int_{\Omega_{0}}\text{f}(\text{X})\cdot \text{u}\;\text{d}\text{X}-\int_{\Gamma_{N,0}}\text{h}(p_{\text{ext}},\text{u}).\text{u}\;\text{d}s_{X}, \end{array}$$

over all admissible fields ***u*** with ***u*** = ***g***_D_ on *Γ*_D_,_0_, where, *Ψ* denotes the strain energy function; *ρ*_0_ denotes the material density in reference configuration; *f* denotes a volumetric body force; ***g***_D_ denotes a given boundary displacement; and ***h*** denotes a given follower load [56,57] defined as

$$\text{h}(p_{\text{ext}},\text{u})≔-p_{\text{ext}}J(\text{u}){\text{F}}^{-⊤}(\text{u})\text{N},$$

with given external constant surface pressure *p*_ext_ ∈ ℝ^+^. Note that in general *p*_ext_ is assumed constant over *Γ*_N,0_ but may vary over time. For ease of presentation it is assumed that *ρ_0_* is constant and ***f***, and ***g***_D_ do not depend on ***u***. For changes of the presented formulation to model transient simulations including inertial terms see [58]. Further, for a discussion of the existence of infimizers in the case of follower loads see the pioneering works of Ball [56],Ciarlet [57].

In this study, we consider nearly incompressible materials, meaning that *J* ≈ 1. A possibility to model this behavior was originally proposed by Flory [23] using a split of the deformation gradient ***F*** – hence referred to as *Flory split* – such that

(2) $$F=F_{\text{vol}}\bar{F}.$$

Here, ***F***_vol_ describes the volumetric change while $\bar{\text{F}}$ describes the isochoric change. By setting ${\text{F}}_{\text{vol}}≔J^{\frac{1}{3}}\text{I}\;\text{and}\;\bar{\text{F}}≔J^{-\frac{1}{3}}\text{F}$ we get det($\bar{\text{F}}$) = 1 and det(***F***_vol_) = *J*. Analogously, by setting ${\text{C}}_{\text{vol}}≔J^{\frac{2}{3}}\text{I}\;\text{and}\;\bar{\text{C}}≔J^{-\frac{2}{3}}\text{C},$ we have ***C*** = ***C***_vol_$\bar{\text{C}}.$ Assuming a hyperelastic material, the Flory split also postulates an additive decomposition of the strain energy function

(3) $$\Psi =\Psi \left(C\right)=U\left(J\right)+\bar{\Psi }\left(\bar{C}\right).$$

The function *U* (*J*) will be used in the form

(4) $$U\left(J\right)≔\frac{\kappa }{2}\Theta {\left(J\right)}^{2}$$

where *κ* > 0 denotes the *bulk modulus.* In the literature many different choices for the functions *Θ*(*J*) are proposed, see e.g [59–61] for examples and related discussion. We want to emphasize that for all choices of *Θ* it holds that *Θ*(1) = 0 and *Θ*'(1) = 1. For studying also the limit case *κ* → ∞ we will consider a reformulation of Eq. (1) as perturbed Lagrangian-multiplier (PL) functional, see [62–67] as well as [68, Chapter 8] for details. Introducing the pressure *p* we seek stationary points (***u***, *p*) ∈ *V*_*g*D_ × *Q* of

(5) $$\begin{array}{l} \Pi^{\text{tot}}\left(\text{u},p\right)≔\Pi^{\text{PL}}\left(\text{u},p\right)+\Pi^{\text{ext}}\left(\text{u}\right),\\ \Pi^{\text{PL}}\left(\text{u},p\right)\;≔\int_{\Omega_{0}}\bar{\Psi }\left(\bar{C}\right)+p\Theta \left(J\left(\text{u}\right)\right)-\frac{1}{2\kappa }p^{2}\text{d}X. \end{array}$$

The above PL formulation is a special case assuming the form (4) for *U*(*J*), see also [63] and [39, Section 3.3]. Variable *p* is not the physical hydrostatic pressure *P*_hydro_ but corresponds to the *applied hydrostatic pressure* [69, Chapter 7, (7.4.44)]. The two pressures are linked by

$$P_{\text{hydro}}=-p\Theta^{\prime }\left(J\right),$$

showing that in the incompressible limit the absolute values of both coincide. To guarantee well-definedness, we assume that

$$V_{g_{\text{D}}}≔\left\{v\in {\left[H^{1}\left(\Omega_{0}\right)\right]}^{3}:v|\Gamma_{D,0}=g_{\text{D}}\right\},$$

with *H*^1^(*Ω*_0_) being the standard Sobolev space of square integrable functions having a square integrable gradient, and *Q* = *L*^2^(*Ω*_0_). For a more in-depth discussion we refer to [56,57]. To solve for stationary points of Eq. (5) we calculate the variations with respect to test functions ***v*** and *q*. This results in the following non-linear variational problem, find (***u***, *p*) ∈ *V*_*g*D_ × *Q* such that

(6) $$R_{\text{vol}}\left(\text{u},p;\text{v}\right)=0,$$

(7) $$R_{\text{inc}}\left(\text{u},p;q\right)=0,$$

for all (***v***, *q*) ∈ *V*_0_ × *Q*. Here,

$$\begin{array}{l} R_{\text{vol}}\left(\text{u},p;\text{v}\right)≔a_{\text{isc}}\left(\text{u};\text{v}\right)+a_{\text{vol}}\left(\text{u},p;\text{v}\right)-l_{\text{fol}}\left(p_{\text{ext}},\text{u};\text{v}\right),\\ R_{\text{inc}}\left(\text{u},p;\text{v}\right)≔b_{\text{vol}}\left(\text{u};q\right)-c(p,q), \end{array}$$

where

$$\begin{array}{lll} a_{\text{isc}}\left(\text{u};\;v\right) & ≔ & \int_{\Omega_{0}}S_{\text{isc}}\left(\text{u}\right):\Sigma \left(\text{u},v\right)\text{d}X,\\ a_{\text{vol}}\left(\text{u},\;p;\;v\right) & ≔ & \int_{\Omega_{0}}pS_{\text{vol}}\left(\text{u}\right):\Sigma \left(\text{u},\;v\right)\text{d}X,\\ b_{\text{vol}}\left(\text{u};\;q\right) & ≔ & \int_{\Omega_{0}}\Theta \left(J\left(\text{u}\right)\right)q\;\text{d}\text{X},\\ c\left(p,\;q\right) & ≔ & \frac{1}{\kappa }\int_{\Omega_{0}}pq\;\text{d}\text{X},\\ l_{\text{fol},\text{N}}\left(P_{\text{ext}},\;\text{u};\;\text{v}\right) & ≔ & \int_{\Gamma_{\text{N},0}}\text{h}\left(P\text{ext},\;\text{u}\right)\;\cdot \;\text{v}\;\text{d}s_{X}, \end{array}$$

with ***Σ***(***u, v***) ≔ sym(***F***^⊤^(***u***)Grad ***v***). Components of the second Piola–Kirchhoff stress tensor

(8) $${\text{S}}_{\text{p}}=p{\text{S}}_{\text{vol}}+{\text{S}}_{\text{isc}}$$

are computed as

$${\text{S}}_{\text{isc}}\;≔\;J^{-\frac{2}{3}}\text{Dev}\left(\bar{\text{S}}\right),\;\bar{\text{S}}\;≔\;2\frac{\partial \bar{\Psi }\left(\bar{\text{C}}\right)}{\partial \bar{\text{C}}}\;{\text{S}}_{\text{vol}}\;≔\;\pi \left(J\right){\text{C}}^{-1},\;\pi \left(J\right)\;≔J\Theta \prime \left(J\right),$$

with Dev(•) being the deviatoric operator in the Lagrangian description, see Eq. (C.1). When modeling electrically active tissue, we consider an additive decomposition of the isochoric part of the stress tensor. The total stress tensor is now given by the additive decomposition

(9) $${\text{S}}_{\text{tot}}={\text{S}}_{\text{a}}+{\text{S}}_{\text{p}}={\text{S}}_{\text{a}}+2\frac{\partial \Psi \left(\text{C}\right)}{\partial \text{C}},$$

with the active stress ***S***_a_ defined as in [70].

To simulate the effect of the circulatory system, these equations are coupled to a 0D lumped model as in [71]. The corresponding nonlinear variational problem reads as find (***u***, *p*) ∈ *V*_*g*_D__ × *Q* and ${\underline{p}}_{\text{CAV}}\in {\mathbb{R}}^{n_{\text{CAV}}}$ such that

(10) $$R_{\text{vol}}\left(\text{u},\;p,\;{\underline{p}}_{\text{CAV}};\;\text{v}\right)=0,$$

(11) $$R_{\text{inc}}\left(\text{u},p;q\right)=0,$$

(12) $$R_{\text{CAV}}\left(\text{u},\;p_{\text{CAV},i}\right)=0\;i=1,\ldots ,n_{\text{CAV},}$$

for all (***v***, *q*) ∈ *V*_0_ × *Q*, and *i* = 1, …, *n*_CAV_, where ${\underline{p}}_{\text{CAV}}=\{p_{\text{CAV},1},p_{\text{CAV},2},\ldots p_{\text{CAV}},n_{\text{CAV}}\}$ denote the cavity pressures, and *n*_CAV_ denotes the number of cavities in the model. Here, the variations read as

(13) $$\begin{array}{lll} R_{\text{vol}}(\text{u},p,{\underline{p}}_{\text{CAV}};\text{v}) & ≔ & a_{\text{isc}}(\text{u};\text{v})+a_{\text{vol}}(\text{u};\text{v})+l_{{\text{fol},\text{CAV}}_{i}}(P_{\text{CAV},i},\text{u};\text{v}),\\ R_{\text{inc}}(\text{u},p;\text{v}) & ≔ & b_{\text{vol}}(\text{u},q)-c(p,q),\\ R_{\text{CAV}}(\text{u},p_{\text{CAV},i}) & ≔ & V_{\text{CAV},i}(\text{u})-V_{\text{CS}}(P_{\text{CAV},i})\;i=1,\ldots ,n_{\text{CAV}}, \end{array}$$

assuming summation over double occurring indices in (13), where

$$V_{\text{CAV},i}(\text{u})≔\frac{1}{3}\int_{\Gamma_{\text{CAV},i,0}}J(\text{X}+\text{u})\cdot {\text{F}}^{-⊤}\text{N}\;\text{d}s_{\text{X}},$$

with *Γ*_CAV,*i*,0_ denoting the closed surface of the *i*th cavity in reference configuration. The expression for cavity volume *V*_CAV,*i*_ follows from applying Nanson’s formula to the definition of cavity volume in the current configuration

(14) $$V_{\text{CAV},i}≔\frac{1}{3}\int_{\Gamma_{\text{CAV},i}}\text{x}\cdot \text{n}\;\text{d}s_{\text{x}}.$$

For a more detailed account on the coupling of nonlinear elastic equations with 0D lumped parameter models, and the computation of *V*_CS_, the volume as predicted by the 0D model of the circulatory system for the intra-cavitary pressure *p*_CAV,*i*_, we refer to [71].

### 2.2 Consistent linearization

In the subsequent section we will use •^*k*^ to indicate a quantity that depends on a previous Newton iterate. For the subsequent discretization we need the consistent linearization of (6)–(7) and (10)–(12) and we obtain the following linear saddle-point problem: for each (***u***^*k*^, *p^k^*) ∈ *V*_*g*_D__ × *Q*, find increments (*Δ**u***, *Δp*) ∈ *V*_0_ × *Q* such that

(15) $$a^{k}\left(\Delta \text{u},\Delta v\right)+a_{\text{N}}^{k}\left(p_{\text{ext}};\Delta \text{u},\Delta v\right)+b^{k}\left(\Delta p,\Delta v\right)=-R_{\text{vol}}\left({\text{u}}^{k},p^{k};\Delta v\right),$$

(16) $$b^{k}\left(\Delta q,\Delta \text{u}\right)-c\left(\Delta p,\Delta q\right)=-R_{\text{inc}}\left({\text{u}}^{k},p^{k};\Delta q\right),$$

where

(17) $$\begin{array}{lll} a^{k}\left(\Delta \text{u},\Delta v\right) & ≔ & \int_{\Omega_{0}}\text{Grad}\;\Delta vS_{\text{tot}}^{k}:\text{Grad}\;\Delta \text{u}\;\text{d}X+\int_{\Omega_{0}}\Sigma \left({\text{u}}^{k},\Delta v\right):{\mathbb{C}}_{\text{tot}}^{k}:\Sigma \left({\text{u}}^{k},\Delta \text{u}\right)\text{d}X,\\ a_{N}^{k}\left(p_{\text{ext}};\Delta \text{u},\Delta v\right) & ≔ & p_{\text{ext}}\int_{\Gamma_{N,0}}J^{k}\left({\left(F^{k}\right)}^{-⊤}:\text{Grad}\;\Delta \text{u}\right)\Delta v\cdot {\left(F^{k}\right)}^{-⊤}N\;\text{d}s_{X}\\ & - & p_{\text{ext}}\int_{\Gamma_{N,0}}J^{k}{\left(F^{k}\right)}^{-⊤}{\left(\text{Grad}\;\Delta \text{u}\right)}^{⊤}{\left(F^{k}\right)}_{k}^{-⊤}N\cdot \Delta v\;\text{d}s_{X,}\\ b^{k}\left(\Delta p,\Delta v\right) & ≔ & \int_{\Omega_{0}}\Delta p\pi \left(J^{k}\right){\left(F^{k}\right)}^{-⊤}:\text{Grad}\;\Delta v\;\text{d}X, \end{array}$$

using the following abbreviations

$$\begin{array}{cc} \begin{array}{lll} F^{k} & ≔ & F\left({\text{u}}^{k}\right),\\ S_{\text{tot}}^{k} & ≔ & S_{\text{isc}}{|}_{\text{u}={\text{u}}^{k}}+P^{k}S_{\text{vol}}{|}_{\text{u}={\text{u}}^{k}},\\ {\mathbb{C}}_{\text{vol}} & ≔ & k\left(J\right)C^{-1}⊗C^{-1}-2\pi \left(J\right)C^{-1}⊙C^{-1}, \end{array} & \begin{array}{lll} J^{k} & ≔ & \text{det}\left(F^{k}\right),\\ {\mathbb{C}}_{\text{tot}}^{k} & ≔ & {\mathbb{C}}_{\text{isc}}{|}_{\text{u}={\text{u}}^{k}}+p^{k}{\mathbb{C}}_{\text{vol}}{|}_{\text{u}={\text{u}}^{k}},\\ k\left(J\right) & ≔ & J^{2}\Theta^{\prime \prime }\left(J\right)+J\Theta^{\prime }\left(J\right), \end{array} \end{array}$$

and ℂ_isc_ given in (C.3). For the deviation of term (17) see Appendix A, other terms in (15)–(16) have been discussed previously, see [58]. Note, that by choosing *U*(*J*) as $\frac{1}{2}$ *Θ*^2^(*J*) we automatically obtain – in the absence of follower loads – a symmetric system, similar to [66]. However, due to the PL formulation we can avoid issues when *U*″(*J*) = 0.

In the case of an attached circulatory system we obtain the following linearized system: find increments $(\Delta \text{u},\Delta p,\Delta {\underline{p}}_{\text{CAV}})\in v_{0}\times Q\times {\mathbb{R}}^{n_{\text{CAV}}}$ such that

(18) $$a^{k}\left(\Delta \text{u},\Delta v\right)+a_{\text{CAV},i}^{k}\left(p_{\text{CAV},i}^{k};\Delta \text{u},\Delta v\right)+b^{k}\left(\Delta p,\Delta v\right)+l_{\text{fol},\text{CAV},i}\left(\Delta p_{\text{CAV},i},{\text{u}}^{k};\Delta v\right)=-R_{\text{vol}}\left({\text{u}}^{k},p^{k},{\underline{p}}_{\text{CAV}}^{k};\Delta v\right),$$

(19) $$b^{k}\left(\Delta q,\Delta \text{u}\right)-c\left(\Delta p,\Delta q\right)=-R_{\text{inc}}\left({\text{u}}^{k},p^{k};\Delta q\right),$$

(20) $$d_{\text{CAV},i}\left({\text{u}}^{k};\Delta \text{u}\right)-e_{\text{CAV},i}\left(p_{\text{CAV},i}^{k};\Delta p_{\text{CAV},i}\right)=-R_{\text{CAV}}\left({\text{u}}^{k},p_{\text{CAV},i}^{k}\right),$$

where

(21) $$e_{\text{CAV},i}\left(p_{\text{CAV},i}^{k};\Delta p_{\text{CAV}}\right)≔\frac{\partial V_{\text{CAV},i}}{\partial p_{\text{CAV},i}},$$

and *d*_CAV_ defined as in (A.3). The term (21) depends on the chosen model for the circulatory system and a detailed discussion is out of the scope of this work. For a detailed derivation of the explicit representation of the compliance matrix (21) stemming from the model used in Section 3 we refer to [71].

### 2.3 Finite element discretization

Here we provide a summary of the FE discretization used in the subsequent results. The framework builds upon methods previously introduced for isotropic, passive mechanics in [58]. In the following, we extend this approach to anisotropic tissues also allowing for complex EM simulations that are coupled to a 0D system of the circulatory system.

Let *𝒯_h_* be a FE partitioning of $\bar{\Omega }$ consisting of isoparametric tetrahedral and/or hexahedral elements. We assume standard regularity assumptions [57] and invertibility of the isoparametric mapping *F_K_* from the reference element $\hat{K}$ to a physical element *K* ∈ *𝒯_h_*. For tetrahedral elements this poses no additional restrictions, for hexahedral elements we refer to [72] for details. Let further ${\hat{P}}_{1}\;\text{and}\;{\hat{Q}}_{1}$ denote the space of lowest order linear/trilinear FE functions on the reference tetrahedron/hexahedron. The discrete analogue to (15)–(16) reads as: given $({\text{u}}_{h}^{k},p_{h}^{k})\in V_{h,g_{\text{D}}}\times Q_{h},$ find (*Δ**u**_h_*, *Δp_h_*) ∈ *V*_*h*,0_ × *Q_h_* such that

(22) $$a^{k}\left(\Delta {\text{u}}_{h},v_{h}\right)+a_{N}^{k}\left(p_{\text{ext}};\Delta {\text{u}}_{h},v_{h}\right)+b^{k}\left(p_{h},v_{h}\right)=-R_{\text{vol}}\left({\text{u}}_{h}^{k},\;p_{h}^{k};\;v_{h}\right),$$

(23) $$b^{k}\left(q_{h},\Delta {\text{u}}_{h}\right)-c\left(\Delta p_{h},q_{h}\right)=-R_{\text{inc}}\left({\text{u}}_{h}^{k},p_{h}^{k};q_{h}\right)$$

for all (***v**_h_*, *q_h_*) ∈ *V*_*h*,0_ × *Q_h_*. The discrete spaces *V*_*h,g*_D__ and *Q_h_* are defined as

$$\begin{array}{c} V_{h,g_{D}}≔\left\{v\in V_{g_{D}}:v{|}_{K}=\hat{v}\circ F_{K}^{-1},\hat{v}\in {\left[\hat{V}\right]}^{3},\forall K\in T_{h}\right\}\;,\\ \;Q_{h}≔\left\{q\in L^{2}\left(\Omega_{0}\right):q{|}_{K}=\hat{q}\circ F_{K}^{-1},\hat{q}\in \hat{\mathbb{Q}},\forall K\in T_{h}\right\}\;, \end{array}$$

with 𝕍 and ℚ denoting suitable spaces of polynomials which will be specified in the subsequent sections.

#### 2.3.1 Pressure projection stabilized equal order pair

The pressure projection stabilization was originally introduced for solving Stokes problems [55] and has also been applied in the context of linear elasticity [73,74]. Recently, we extended its use to isotropic, nonlinear elasticity [58]. A similar approach can be used for anisotropic materials, we set $\hat{𝕍}≔\hat{\mathbb{Q}}≔{\hat{P}}_{1}/{\hat{Q}}_{1}$ for tetrahedral or hexahedral elements. To ensure stability, we have to modify the definition of the residuals in (7) and (11) to

(24) $$\begin{array}{lll} {\tilde{R}}_{\text{inc}}\left({\text{u}}_{h},\;p_{h};\;q_{h}\right) & ≔ & R_{\text{inc}}\left({\text{u}}_{h},\;p_{h};\;q_{h}\right)-s_{h}\left(p_{h},\;q_{h}\right),\\ s_{h}\left(p,q\right) & ≔ & \int_{\Omega_{0}}\frac{1}{\mu_{*}}\left(p-\Pi_{h}p\right)\left(q-\Pi_{h}q\right)\text{d}x, \end{array}$$

where the projection operator *Π_h_* is defined elementwise as

$$\Pi_{h}q{|}_{K}≔\frac{1}{\left|K\right|}\int_{K}q\;\text{d}x.$$

In contrast to [58], the parameter *μ*_∗_ is no longer an arbitrary value but set to |*K*|^1/3^; a choice that showed excellent results for all discussed anisotropic problems in Section 3 as well as isotropic benchmarks in [58]. We note, that the integral in (24) has to be understood as a sum over all the elements of the triangulation of the domain *Ω*_0_. For a more comprehensive overview and implementation details we refer to [58].

#### 2.3.2 MINI element

**Fig. 1:**
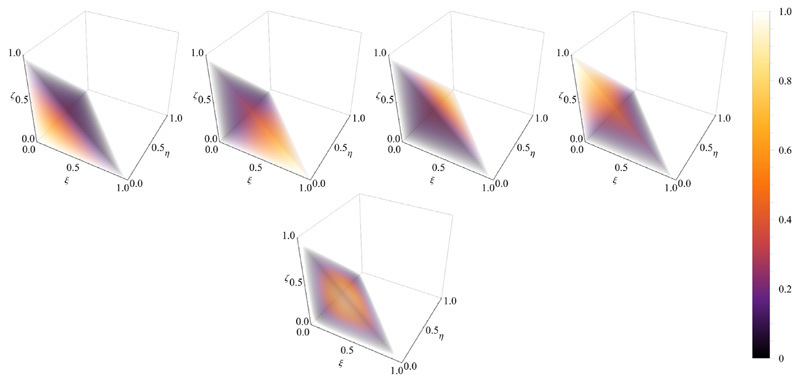
Ansatz functions First row shows the standard linear basis functions over the reference tetrahedron, second row shows the bubble function.

**Fig. 2:**
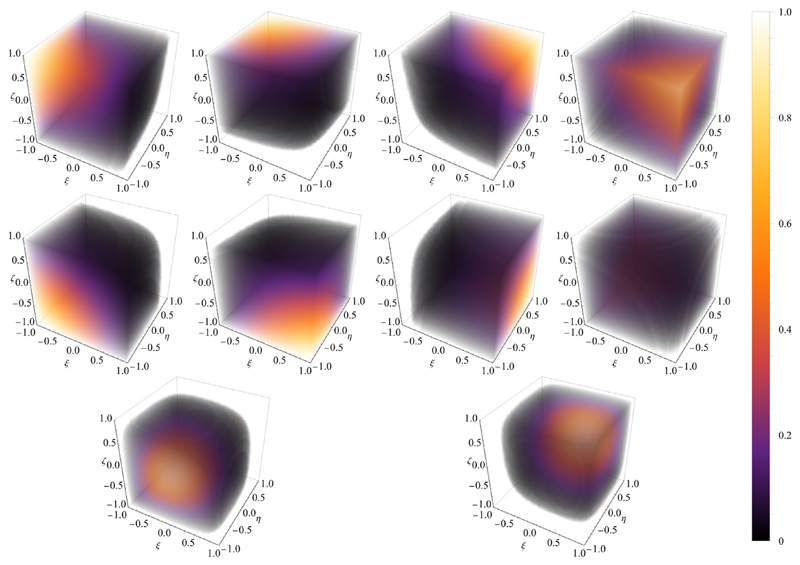
Ansatz functions First two rows show the standard trilinear basis functions over the reference hexahedron, and the third row shows the two bubble function.

One of the earliest strategies in constructing a stable FE pairing for discrete saddle-point problems arising from Stokes equations is the MINI element, dating back to the works of Brezzi et al. see for example [52,75]. Briefly, the strategy is to enrich the basis of lowest order elements by adding a higher degree polynomial with support restricted to the interior of the element. Thus, for the tetrahedral reference element $\hat{K}$ we define

$$\begin{array}{lll} \hat{V} & ≔ & {\hat{P}}_{1}⊕\left\{{\hat{\Psi }}_{\text{B}}\right\}\\ \hat{\mathbb{Q}} & ≔ & {\hat{P}}_{1},\\ {\hat{\Psi }}_{\text{B}} & ≔ & 256\;\xi \eta \zeta \;\left(1-\xi -\eta -\zeta \right), \end{array}$$

where (*ξ, η, ζ*) ∈ $\hat{K}$ see also [76]. For the hexahedral reference element $\hat{K}={\left[-1,1\right]}^{3}$ we define

(25) $$\begin{array}{l} \;\hat{V}≔{\hat{Q}}_{1}⊕\left\{{\hat{\Psi }}_{\text{B},1},{\hat{\Psi }}_{\text{B},2}\right\}\\ \;\hat{\mathbb{Q}}≔{\hat{Q}}_{1},\\ {\hat{\Psi }}_{\text{B},1}≔\left(1-\xi^{2}\right)\left(1-\eta^{2}\right)\left(1-\zeta^{2}\right){\hat{\Psi }}_{\alpha },\\ {\hat{\Psi }}_{\text{B},2}≔\left(1-\xi^{2}\right)\left(1-\eta^{2}\right)\left(1-\zeta^{2}\right){\hat{\Psi }}_{\beta }, \end{array}$$

for (*ξ, η, ζ*) ∈ $\hat{K}$ and (*α*, *β*) ∈ [1, 8] denoting the indices of two ansatz functions for diagonal opposite nodes in $\hat{K},$ see [58]. The choice of the two numbers *α*, *β* depends on the nodal ordering of the reference hexahedron. A visualization of the ansatz functions used in this work is given in Figs. 1 and 2.

Classical results [76] guarantee the stability of the MINI element for tetrahedral meshes in the almost incompressible linear elastic case. For hexahedral elements we were able to prove stability in the almost incompressible linear elasticity case provided an enrichment like (25) of the displacement ansatz space by two bubble functions see [58]. Due to the compact support of the bubble functions, static condensation can be applied to remove the interior degrees of freedom during assembly. Static condensation can be done by standard procedures [76] with the exception of follower loads which is discussed in Appendix B. As a result, these degrees of freedom are not needed to be considered in the full global stiffness matrix assembly which is a key advantage of the MINI element.

### 2.4 Material models

Arterial and myocardial tissue as modeled in Section 3 is considered as a non-linear, hyperelastic, nearly incompressible, and anisotropic material with a layered organization of fibers. To model this behavior in our benchmark problems we used strain energy functions of the form (3). First, *single Fung-type exponential models* of the form

(26) $$\Psi \left(\text{C}\right)=\frac{\kappa }{2}\Theta {\left(J\right)}^{2}+\bar{\Psi }\left(\bar{\text{C}}\right)\;\text{with}\;\bar{\Psi }\left(\bar{\text{C}}\right)-\frac{a}{2}\left[\exp Q\left(\bar{\text{C}},a_{i}\right)-1\right]$$

with *a* > 0kPa being a scaling parameter, ***a**_i_* are fiber directions, and 𝒬 is a function in terms of scalar strain components. The specific form of *U*(*J*) and 𝒬 will be discussed later in the benchmark section.

Second, *separated, invariant-based Fung-type exponential models*, also often referred to as *Holzapfel–Ogden-type models.* Here, we compared the standard formulation – using anisotropic splitting (AS) –

(27) $$\Psi \left(\text{C}\right)=\frac{\kappa }{2}\Theta {\left(J\right)}^{2}+{\bar{\Psi }}_{\text{AS}}\left(\bar{\text{C}}\right)\;\text{with}\;{\bar{\Psi }}_{\text{AS}}\left(\bar{\text{C}}\right)={\bar{\Psi }}_{\text{iso}}\left(\bar{\text{C}}\right)+{\bar{\Psi }}_{\text{aniso}}\left(\bar{\text{C}},a_{i}\right)$$

with the formulation using an unsplit deformation gradient for the anisotropic contribution – without anisotropic split (WAS) –

(28) $$\Psi \left(\text{C}\right)=\frac{\kappa }{2}\Theta {\left(J\right)}^{2}+\Psi_{\text{WAS}}\left(\text{C}\right)\;\text{with}\;\Psi_{\text{WAS}}\left(\text{C}\right)={\bar{\Psi }}_{\text{iso}}\left(\bar{\text{C}}\right)+{\bar{\Psi }}_{\text{aniso}}(\text{C},a_{i}),$$

introduced in [77,78]. The specific form of the volumetric, *U*(*J*), isotropic, ${\bar{\Psi }}_{\text{iso}},$ and anisotropic, ${\bar{\Psi }}_{\text{aniso}}/\Psi_{\text{aniso}},$ contributions will be discussed later for each of the benchmark problems.

Note that results on existence of solutions in nonlinear elasticity as well as stability of fiber-reinforced materials are highly dependent on polyconvex strain-energy functions. This is not guaranteed for above mentioned models, however, we use the same models and parameters for our benchmark problems as reported in previous works, i.e., the setup from Gültekin et al. [79] for the benchmark in Section 3.1 and the setup from Land et al. [80] for the benchmark in Section 3.2. For the third numerical example in Section 3.3 we use a state-of-the-art Holzapfel–Ogden type material which are most commonly used to describe passive tissue mechanics.

For more details regarding the existence of solutions and the stability of the material models we refer to [56,57,78,81,82].

## 3 Numerical examples

Biomechanical applications often require highly resolved meshes and thus efficient and massively parallel solution algorithms for the linearized system of equations become an important factor to deal with the resulting computational load. Extending our previous implementations for cardiac electro-mechanics (EM) [83] we implemented the stabilization techniques in the FE framework Cardiac Arrhythmia Research Package *(CARPentry)* [84,85], built upon extensions of the *openCARP* EP framework [86] (http://www.opencarp.org). We solve the stabilized saddle-point problem (22)–(23) by using a GMRES method with a block preconditioner based on a smoothed aggregation algebraic multigrid (GAMG) approach which is incorporated in *PETSc* [87,88].

In all of the following benchmark problems our goal was to study the performance and accuracy of different FE discretizations, namely (i) *Q1/P1–P0-AS:* discretization with piecewise linear displacements and piecewise constant pressure using the strain energy function (27); (ii) *Q1/P1–P0-WAS:* discretization with piecewise linear displacement and piecewise constant pressure using the strain energy function (28); (iii) *Projection:* equal order discretization with piecewise linear displacements and pressure, stabilized as described in Section 2.3.1 using the strain energy function (27); (iv) *MINI:* discretization using MINI elements as described in Section 2.3.2 using the strain energy function (27).

Meshes for Sections 3.1 and 3.2 were created using Gmsh [89]. Tetrahedral meshes were created as tetrahedralization of the hexahedral mesh and we use *ℓ* to index the various uniform refinement levels of the meshes.

### 3.1 Extension, inflation and torsion of a simplified artery model

#### Simulation setup

**Fig. 3:**
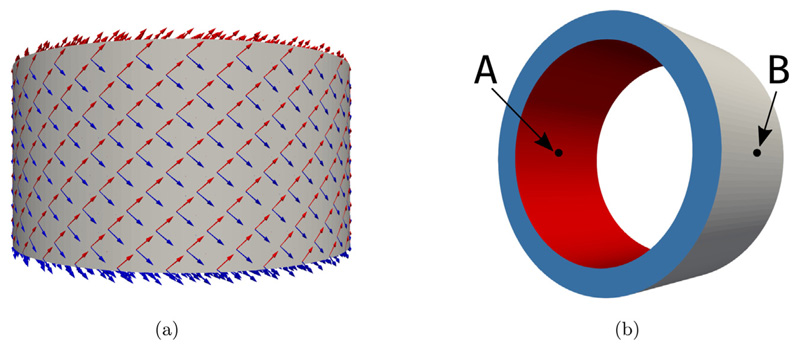
Artery benchmark Geometry, fiber distribution, and boundary conditions. Figure (a) shows the fiber arrangement and Figure (b) shows the geometry and colorcoded boundaries for the application of the boundary conditions and the evaluation points A, and B for mesh convergence.

First, we show the applicability of our proposed methods to a benchmark problem from Gültekin et al. [79] where a simplified artery model is represented by a thick-walled cylindrical tube. The dimensions of this idealized geometry with its centerline on the *z*-axis are as follows: height *H* = 10 mm, inner radius *R*_1_ = 8 mm, and outer radius *R*_2_ = 10 mm. Two symmetric families of fibers, ***f***_0_ and ***s***_0_ are immersed in the tissue, having an angle of 40° with circumferential *θ*-axis, see Fig. 3A. As for loading, a monotonically increasing displacement up to 2mm superimposed by a monotonically increasing torsion up to 60° is applied on the top of the tube (marked blue in Fig. 3B). Additionally, a linearly increasing pressure (follower load) up to 500 mmHg is applied on the inside of the tube (marked red in Fig. 3B). Finally, the lower part of the tube is clamped at zero displacement.

The material is described by the strain-energy function (27), ${\bar{\Psi }}_{\text{AS}},$ with

$$\begin{array}{r} \Theta (J)≔J-1,\;{\bar{\Psi }}_{\text{iso}}(\bar{\text{C}})≔\frac{\mu }{2}({\bar{I}}_{1}-3),\\ {\bar{\Psi }}_{\text{aniso}}(\bar{\text{C}},f_{0},s_{0})≔\frac{k_{1}}{2k_{2}}\sum_{i=4,6}(\exp(k_{2}{\left({\bar{I}}_{i}-1\right)}^{2})-1), \end{array}$$

and invariants

$${\bar{I}}_{1}≔\text{tr}(\bar{\text{C}}),\;{\bar{I}}_{4}≔\bar{\text{C}}:f_{0}⊗f_{0},\;{\bar{I}}_{6}≔\bar{\text{C}}:s_{0}⊗s_{0},$$

and analogously with *Ψ*_aniso_(***C**, **f***_0_, ***s***_0_) for the WAS formulation, *Ψ*_WAS_, (28). Material parameters were taken from [79, Table 1], i.e., *κ* = 5000 kPa, *μ* = 10 kPa, *k*_1_ = 500 kPa, and *k*_2_ = 2.0. In case of the stabilized equal-order elements (Projection and MINI), we set 1/*κ* = 0 to render the material incompressible. To assess mesh convergence simulations were performed on seven discretization levels, see Table 1.

#### Results

**Fig. 4:**
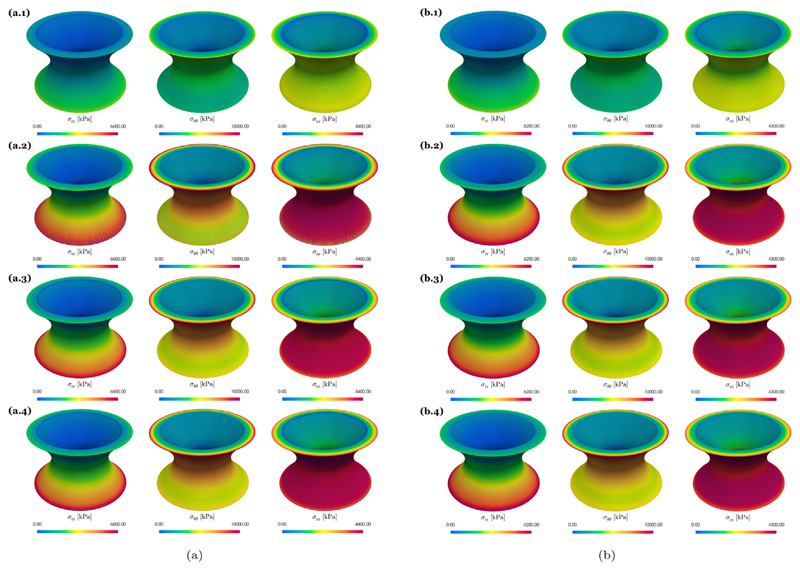
Artery benchmark Stress distribution for (a) tetrahedral and (b) hexahedral elements. Shown are the radial, *σ*_rr_, circumferential, *σ_θθ_*, and longitudinal stresses, *σ_zz_*, on the finest discretization level *ℓ* = 7. The rows 1, 2, 3, 4 correspond to Q1–P0-AS, Q1–P0-WAS, Projection, and MINI, respectively.

**Fig. 5:**
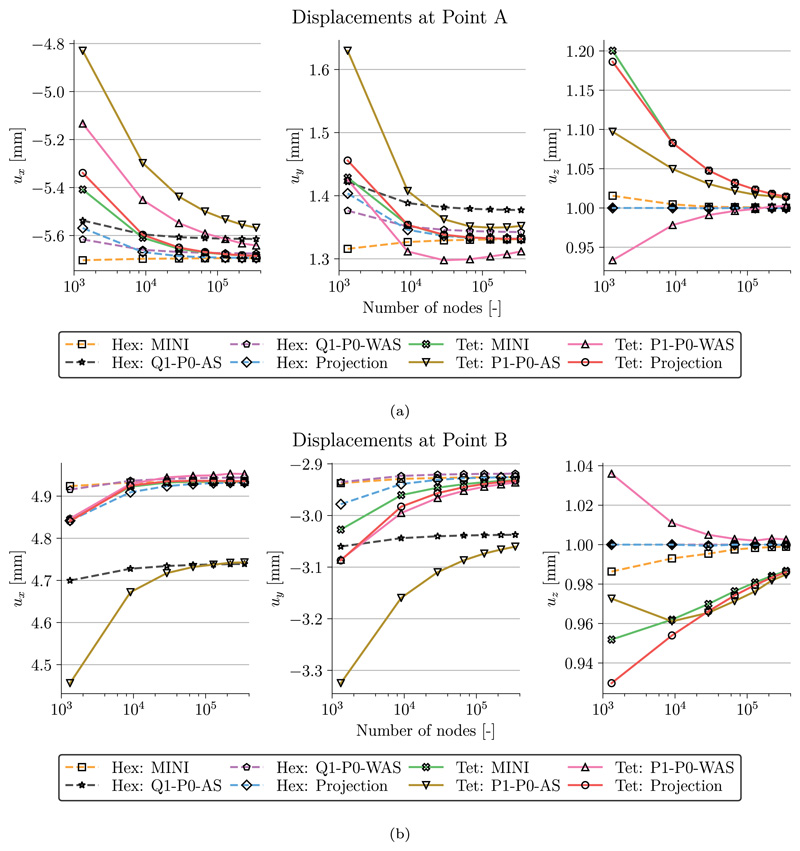
Artery benchmark Mesh convergence. Shown are the individual displacement values at (a) Point A and (b) Point B for increasing mesh resolution and finite elements.

**Fig. 6:**
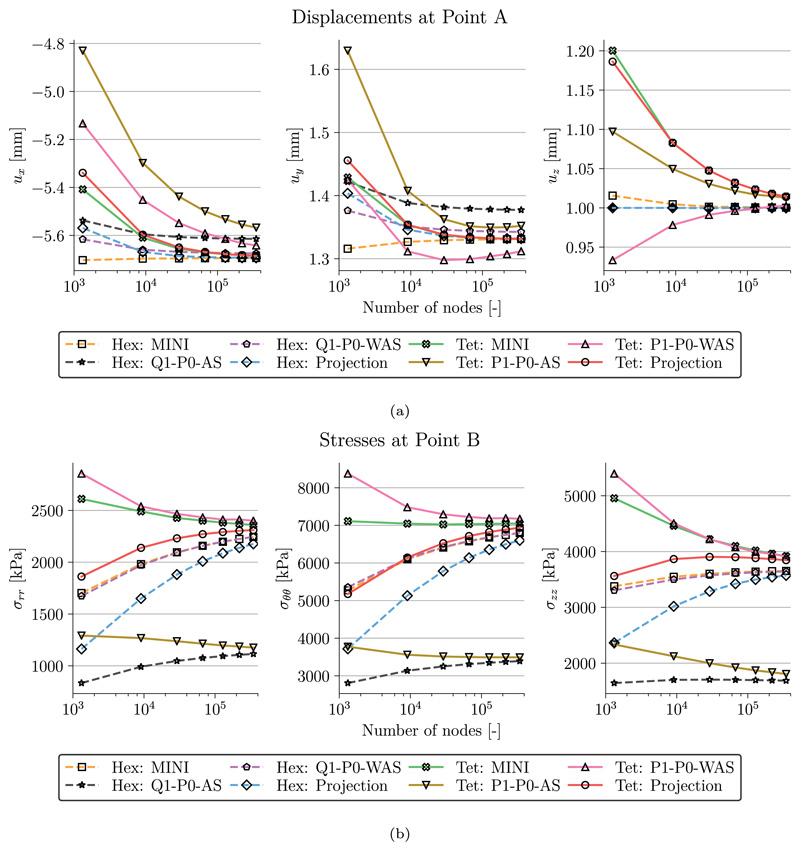
Artery benchmark Mesh convergence. Shown are the individual stresses at (a) Point A and (b) Point B for increasing mesh resolution and finite elements.

A comparison of the radial, *σ*_rr_, the circumferential, *σ_θθ_*, and the axial, *σ*_zz_, components of the Cauchy stress tensor is shown in Fig. 4 for the finest discretization level *ℓ* = 7. We see that with the exception of the lowest order discretizations with anisotropic splitting (Q1/P1–P0-AS) the stress distribution is very similar and also matches results in Gültekin et al. [79]. The observation that simulations with Q1/P1–P0-AS are not accurate for this benchmark problem is further emphasized in Figs. 5 and 6. Here, Fig. 5 shows the displacements (*u_x_, u_y_, u_z_*) and Fig. 6 shows the stress components (*σ*_rr_, *σ_θθ_*, *σ_zz_*) at the evaluation points A and B over the discretization levels. In agreement with [79] the lowest order discretization with anisotropic splitting converges to a lower value than the other discretization types hinting at possible locking phenomena. All other formulations perform similarly well. Here, discrepancies at the finest level *ℓ* = 7 are rather due to differences in the meshes for tetrahedral and hexahedral grids.

**Fig. 7:**
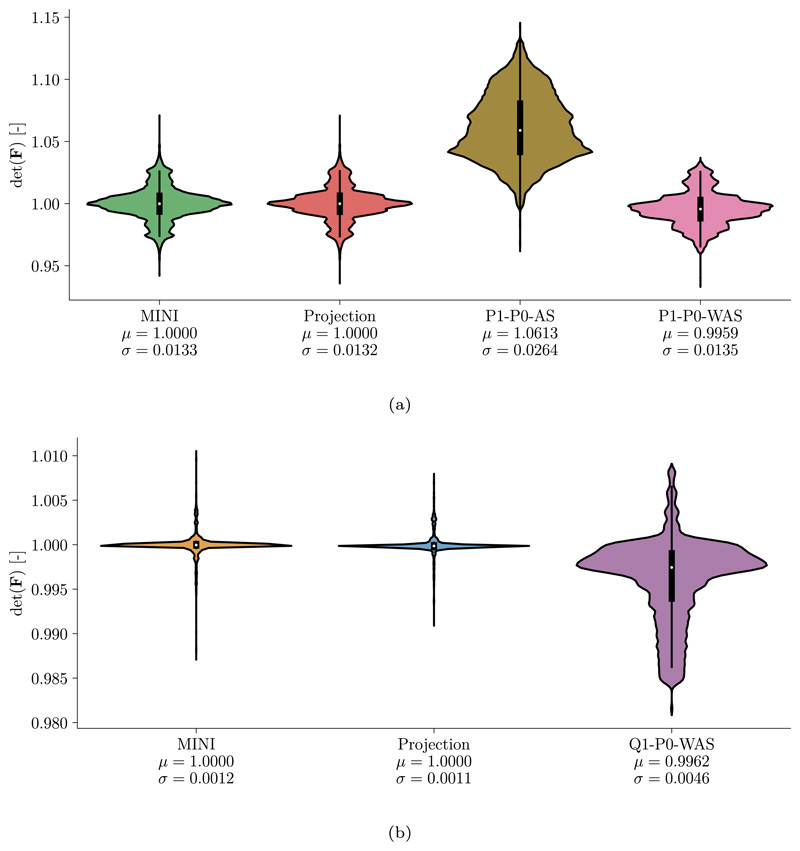
Artery benchmark Jacobian distribution for (a) tetrahedral and (b) hexahedral elements. Shown are violin plots of the Jacobian distribution det(***F***) on the finest discretization level at maximum loading for the various finite elements. Additionally, the mean *μ* and standard deviation *σ* are given. Q1–P0-AS has been excluded from plot (b) as values were significantly higher compared to other element types.

Fig. 7 shows a distribution of the Jacobian det(***F***) on the finest level *ℓ* = 7. Unsurprisingly, with a mean value *μ* close to 1 the saddle-point formulations (Projection, MINI) satisfy incompressibility better than the penalty formulations (P1/Q1–P0-AS, P1/Q1–P0-WAS). While the AS formulation led to a small increase in volume (*μ* > 1), the WAS formulation resulted in a slightly reduced volume (*μ* < 1).

#### Numerical performance

**Fig. 8:**
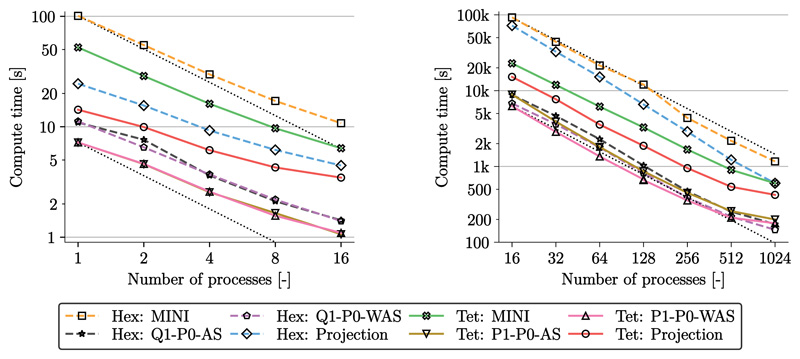
Artery benchmark Strong scaling results for the different element types for the coarsest grid (*ℓ* = 1, left) and the finest grid (*ℓ* = 7, right). Simulations were performed on 1 to 16 cores of a standard desktop computer for the coarse problem and on 16 to 1024 cores on Archer2.

Computational times for the simulation using different element types are given in Fig. 8; left, for the coarse problem (*ℓ* = 1) and right, for the finest grid (*ℓ* = 7). For all cases we used a relative error reduction of *ϵ* = 10^−8^ for the GMRES linear solver and a relative error reduction of *ϵ* = 10^−6^ for the residual of the Newton method. With a load stepping scheme the applied traction and displacement was increased to arrive at the final prescribed displacement and an inner pressure of 500 mmHg. Using 25 loading steps for the coarse problem required a total number of 100 linear solving steps. For the fine problem we chose 250 loading steps and required 1000 linear solving steps. Strong scaling was achieved for the coarse problem up to 16 cores on a desktop machine (AMD Ryzen Threadripper 2990X), see Fig. 8, left. Here, computational times were averaged over 5 runs using the same setup and range from 1 s to 1.5 s for penalty formulations (P1/Q1–P0-AS, P1/Q1–P0-WAS). For the projection-stabilized element compute times were around 3 times slower (Tet Projection: 3.5 s, Hex Projection: 4.5 s) while for the MINI element compute times were around 7 times slower (Tet MINI: 6.4 s, Hex MINI: 10.8 s).

For the fine grid strong scaling was obtained up to 1024 cores on Archer2 (https://www.archer2.ac.uk/), see Fig. 8, right. Computational times using 1024 cores were between 146 s and 200 s for penalty formulations (P1/Q1–P0-AS, P1/Q1–P0-WAS); around 3 times slower for the projection-stabilized elements (Tet Projection: 422 s, Hex Projection: 601 s); and around 6 times slower for MINI elements (Tet MINI: 600 s, Hex MINI: 1166 s).

#### Discussion

In accordance with Gültekin et al. [79] we have shown for this benchmark that the concept Q1/P1–P0-WAS is able to match quasi-incompressible responses compared to the gold standard of locking-free elements. For this extreme loading case standard Q1/P1–P0-AS elements cannot reproduce accurate stress distributions; not even on very fine grids, see Fig. 6(b).

Further, this benchmark highlights the high efficiency of our methods. Computational times of the benchmark as presented in [79] – on a 3.0 GHz CPU unit and the same hexahedral mesh – were 11 s for Q1–P0-WAS elements and 24 s, i.e., only 2 to 3 times slower, for locking-free Hex Projection elements, see Fig. 8, left. Similarly, for tetrahedral elements as well as different refinement levels, simulations are only to a relatively small factor slower when using locking-free elements (Fig. 8). Further, as illustrated in this figure, strong scaling properties were excellent for both: computations using the coarsest grid on a desktop machine, as well as computations using the finest grid on a supercomputer.

Due to a higher number of linear iterations and higher matrix assembly times, simulations with hexahedral meshes were more expensive compared to simulations with tetrahedral grids. However, as also observed, e.g., by Chamberland et al. [90] hexahedral elements were slightly more accurate than their tetrahedral equivalent. In real-world applications the minor gain in accuracy has to be balanced with the higher cost of hex elements in terms of computing and mesh generation when geometrically detailed anatomical models are used.

### 3.2 Inflation and active contraction of a simplified ventricle

#### Simulation setup

To verify our EM setup we repeated the inflation and active contraction benchmark from Land et al. [80]. We generated the reference geometry of an idealized ventricle as a tetrahedral mesh of a truncated ellipsoid and prescribed a local orthonormal coordinate system with fiber, ***f***_0_, sheet, *s*_0_, and sheet-normal, ***n***_0_, directions according to this paper. We constructed three levels of refinement, see Table 2 for discretization details.

As material we used the transversely isotropic law by Guccione et al. [91], based on Eq. (26), with *Θ*(*J*) ≔ ln(*J*) and

$$Q≔b_{\text{f}}{\left(f_{0}\cdot \bar{E}f_{0}\right)}^{2}+b_{\text{t}}\left[{\left(s_{0}\cdot \bar{E}s_{0}\right)}^{2}+{\left(n_{0}\cdot \bar{E}n_{0}\right)}^{2}+2{\left(s_{0}\cdot \bar{E}n_{0}\right)}^{2}\right]+2b_{\text{fs}}\left[{\left(f_{0}\cdot \bar{E}s_{0}\right)}^{2}+{\left(f_{0}\cdot \bar{E}n_{0}\right)}^{2}\right].$$

Constitutive parameters were *a* = 2 kPa, *b*_f_ = 8, *b*_t_ = 2, and *b*_fs_ = 4. In the benchmark paper the material is considered to be fully incompressible, hence, we chose 1/*κ* = 0 for the saddle-point formulation (Projection, MINI). For the penalty formulation (P1–P0 elements) we chose *κ* = 1000 kPa which was the best trade-off between convergence of the solver for all three levels in Table 2, near incompressibility, and minimization of locking effects.

As the material law above does not allow for a WAS formulation we repeated the benchmark using a separated, invariant-based Fung-type exponential model as in Eq. (27). In particular, we chose a Holzapfel–Ogden material [92] of the form

(29) $$\begin{array}{l} \Theta \left(J\right)≔\text{ln}\left(J\right),\;{\bar{\Psi }}_{\text{iso}}\left(\bar{\text{C}}\right)≔\frac{a}{2b}\left\{\text{exp}\left[b\left({\bar{I}}_{1}-3\right)\right]-1\right\}\\ {\bar{\Psi }}_{\text{aniso}}\left(\bar{\text{C}},{\text{f}}_{0},{\text{s}}_{0},{\text{n}}_{0}\right)≔\sum_{i=\text{f},\text{n}}\frac{a_{i}}{2b_{i}}\left\{\text{exp}\left[b_{i}{\left({\bar{I}}_{4i}-1\right)}^{2}\right]-1\right\}+\frac{a_{\text{fs}}}{2b_{\text{fs}}}\left\{\text{exp}\left[b_{\text{fs}}{\left({\bar{I}}_{8\text{fs}}\right)}^{2}\right]-1\right\}, \end{array}$$

with invariants

$${\bar{I}}_{1}≔\text{tr}\left(\bar{\text{C}}\right),\;{\bar{I}}_{4\text{f}}=\text{max}\left({\text{f}}_{0}\cdot {\bar{\text{C}}\text{f}}_{0},1\right),\;{\bar{I}}_{4\text{n}}=\text{max}\left({\text{n}}_{0}\cdot \bar{\text{C}}{\text{n}}_{0},1\right),$$

such that contributions of compressed fibers are excluded, and the interaction-invariant

$${\bar{I}}_{8\text{fs}}=f_{0}\cdot \bar{\text{C}}s_{0}.$$

Analogously, we used the constitutive equation above with *Ψ*_aniso_(***C***, ***f***_0_, ***s***_0_, ***n***_0_) for the WAS formulation. Material parameters were taken from [93], *a* = 0.809 kPa, *b* = 7.474, *a*_f_ = 1.911 kPa, *b*_f_ = 22.063, *a*_n_ = 0.227 kPa, *b*_n_ = 34.802, *a*_fs_ = 0.547 kPa, and *b*_fs_ = 5.691, fitted to human myocardial experiments in [94].

#### Results

**Fig. 9:**
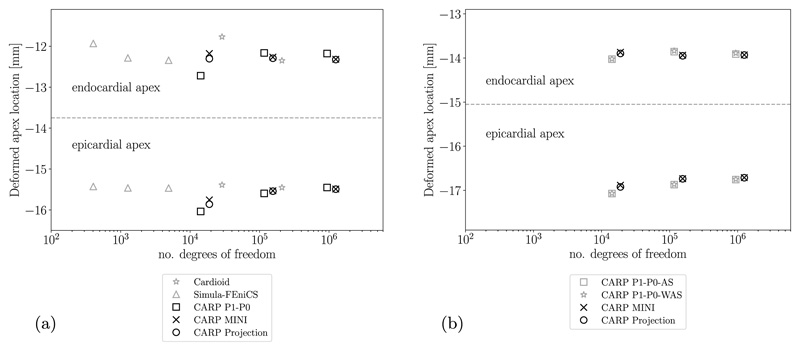
Ellipsoid benchmark apex location. The dashed line separates results for the deformed positions of the apex at the endo- and epicardium. (a) Guccione material with comparison to benchmark results (in gray) presented in [80]; (b) Holzapfel–Ogden material with comparison to P1–P0-WAS formulation.

**Fig. 10:**
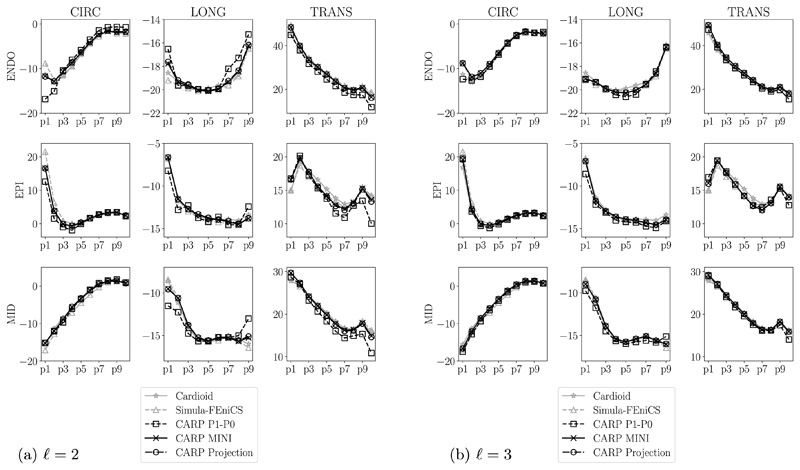
Ellipsoid benchmark, Guccione material longitudinal (LONG), circumferential (CIRC), and radial (TRANS) strains at endocardium, epicardium, and midwall. Index of points increases from the apex to the base. Own results (in black) are compared to benchmark results (in gray) presented in [80].

For the transversely isotropic law (26), we compared our results to selected reference solutions from the benchmark paper [80], namely, the result from IBM with the Cardioid framework [95] using P2–P1 elements and the result from Simula with FEniCS [96] using two-dimensional P2–P1 elements. First, the final location of the apex is measured and, second, circumferential, longitudinal, and radial strains at the endocardium, epicardium, and midwall are calculated on points along apex-to-base lines, see [80] for more details. Results show that the apex location Fig. 9(a) and strains Fig. 10 are very similar for the finest level (*ℓ* = 3) for all chosen element types. For the level *ℓ* = 2 the strain solution using simple P1–P0 elements is not converged showing differences to the benchmark solutions especially in boundary regions at the apex (p1) and the base (p10), see Fig. 10(a).

**Fig. 11:**
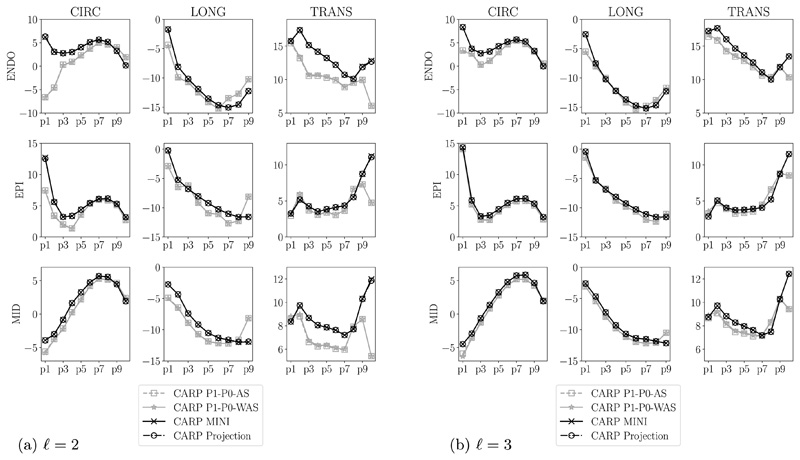
Ellipsoid benchmark, Holzapfel–Ogden material longitudinal (LONG), circumferential (CIRC), and radial (TRANS) strains at endocardium, epicardium, and midwall. Index of points increases from the apex to the base. P1–P0 elements (in gray) are compared to stabilized elements (in black).

We repeated simulations as above measuring the final apex location and calculating strains along apex-to-base lines using the orthotropic law (29). We compared results using P1–P0-WAS, P1–P0-AS, projection-stabilized, and MINI elements in Fig. 9(b) and Fig. 11. Apex locations are very similar for all element types, however, strains are different, especially in boundary regions close to the apex and the base. We can see in Fig. 11 that even for the finest level (*ℓ* = 3) the strain solution for both P1–P0 formulations is not converged while solutions for stabilized elements are already very similar for levels *ℓ* = 2 and *ℓ* = 3.

#### Discussion

For the transversely-isotropic Guccione material model strains with the presented projection-stabilized and MINI elements match results using higher order P2–P1 elements even on coarser grids. Here, also linear tetrahedral elements seem to be accurate given a fine enough discretization; this behavior was also observed in [80].

In contrast, for the orthotropic Holzapfel–Ogden material, we see in Fig. 11 that P1–P0 elements cannot always accurately reproduce strains even on the finest grid. On the other hand we can assume that strains are accurate and almost converged for projection-stabilized and MINI elements as results for levels 2 and 3 are very similar. Interestingly, for this benchmark, we see no difference in accuracy between the standard P1–P0 and the P1–P0-WAS formulation. Most likely the reason for this is that parameters fitted to human myocardial data [93] are not as stiff in fiber direction compared to the more extreme artificial benchmark case in Section 3.1. Overall, both approaches with simple linear elements fail to match results from gold-standard elements, especially in boundary regions.

### 3.3 3D-0D closed-loop model of the heart and circulation

#### Simulation setup

**Fig. 12:**
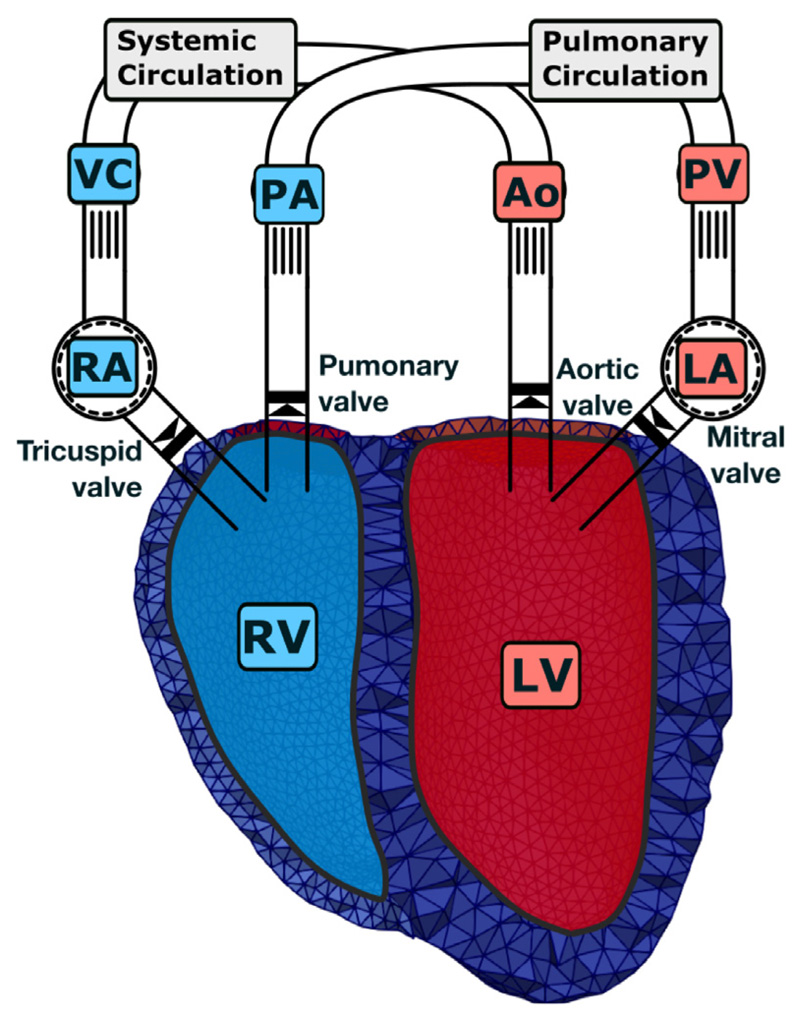
3D–0D model of the heart A 3D PDE model of the ventricles (LV, RV) – represented by the finite element mesh – is coupled to a 0D ODE model of the remainder of the circulatory system. Here, atria (LA, RA) are modeled as lumped cavities; vessels, i.e., aorta (Ao), pulmonary artery (PA), pulmonary vein (PV), and vena cavae (VC) are modeled as lumped tubes; and distal systemic circulation and lungs are modeled as resistances. Valvular opening and closing motion is only modeled in the 0D model while in the 3D model triangulated membranes serve to close the ventricles. Red colors indicate oxygenated and blue colors de-oxygenated blood. For more information on the 3D–0D coupling see [71].

Finally, we show the applicability of our method to an advanced model of computational cardiac EM. Here, a 3D model of bi-ventricular EM is coupled to the physiologically comprehensive 0D CircAdapt model representing atrial mechanics and closed-loop circulation, see Fig. 12. In the present paper, the myocardium of the ventricles was modeled as a nonlinear hyperelastic, (nearly) incompressible and orthotropic material as in Eq. (27). In particular, for this application, we chose the model proposed by Gültekin et al. [97]

$$\begin{array}{l} \;\Theta \left(J\right)≔\text{In}\left(J\right),\;{\bar{\Psi }}_{\text{iso}}\left(\bar{\text{C}}\right)≔\frac{a}{2b}\left\{\exp\left[b\left({\bar{I}}_{1}-3\right)\right]-1\right\}\\ {\bar{\Psi }}_{\text{aniso}}\left(\bar{\text{C}},{\text{f}}_{0},{\text{s}}_{0}\right)≔\sum_{i=\text{f},\text{s}}\frac{a_{i}}{2b_{i}}\left\{\exp\left[b_{i}{\left({\bar{I}}_{4i}-1\right)}^{2}\right]-1\right\}=\frac{a_{\text{f}s}}{2b_{\text{f}s}}\left\{\exp\left[b_{\text{f}s}{\left({\bar{I}}_{8\text{f}s}\right)}^{2}\right]-1\right\}, \end{array}$$

with modified unimodular fourth-invariants to support dispersion of fibers

$$I_{4i}^{*}=\kappa_{i}{\bar{I}}_{1}+\left(1-3\kappa_{i}\right)I_{4i},\;i\in \text{f},\text{s}$$

and standard invariants

$${\bar{I}}_{1}≔\text{tr}\left(\bar{\text{C}}\right),\;{\bar{I}}_{8\text{f}s}={\text{f}}_{0}\cdot {\bar{\text{C}}}_{{\text{s}}_{0}}.$$

Analogously, we used the constitutive equation above with *Ψ*_aniso_(***C***, ***f***_0_, ***s***_0_) for the WAS formulation.

A reaction-eikonal model [85] was used to generate electrical activation sequences. Cellular dynamics were described by the Grandi–Pasqualini–Bers model [98] of human ventricular electrophysiology coupled to the Land–Niederer model [99] of human active stress generation to account for length and velocity dependence of active stress generation, see also Augustin et al. [83] for more details on this strong coupling as well as Regazzoni and Quarteroni [100] for implementation details on the velocity-dependent active stress model. The active stress tensor is computed according to [101] as

$${\text{S}}_{\text{a}}=S_{\text{a}}\left(\frac{\kappa_{\text{f}}}{1-2_{\kappa_{\text{f}}}}{\text{C}}^{-1}+\frac{1-3\kappa_{\text{f}}}{1-2_{\kappa_{\text{f}}}}{\left({\text{f}}_{0}\cdot \text{C}{\text{f}}_{0}\right)}^{-1}{\text{f}}_{0}⊗{\text{f}}_{0}\right),$$

where *S*_a_ is the scalar valued active stress generated by the cardiac myocytes and *κ*_f_ is the same dispersion parameter as above.

For the time integration of Cauchy’s equation of motion we used a variant of the generalized-*α* integrator [102] with spectral radius *ρ*_∞_ = 0 and damping parameters *β*_mass_ = 0.1 ms^−1^, *β*_stiff_ = 0.1 ms.

#### Bi-ventricular finite element models

The bi-ventricular geometry was created according to [71] with an average spatial resolution of 1.3 mm for the LV and 1.2mm for the RV. The resulting mesh used for simulations consisted of 557 316 tetrahedral elements and 111234 nodes. Fiber and sheet directions were computed by a rule-based method [103] with fiber angles changing linearly from −60° at the epicardium to +60° at the endocardium [104]. The valve openings were closed by solid lids as described in [71] to generate the closed surfaces as needed for Eq. (14).

#### Boundary conditions

Boundary conditions on the epicardium were modeled using spatially varying normal Robin boundary conditions [105] to simulate *in-vivo* constraints imposed by the pericardium. The basal cut plane was constrained by omni-directional spring type boundary conditions. The 3D ventricular PDE model was coupled to the 0D ODE model *CircAdapt* [106] representing cardiovascular system dynamics according to Augustin et al. [71]. An *ex-vivo* setting without pericardial boundary conditions is used to calibrate parameters replicating *ex-vivo* passive inflation experiments by Klotz et al. [107].

#### Parameterization

Passive material parameters *a* = 0.4, *b* = 6.55, *a*_f_ = 3.05, *b*_f_ = 29.05, *a*_s_ = 1.25, *b*_s_ = 36.65, *a*_fs_ = 0.15, and *b*_fs_ = 6.28 were taken from [97]. Dispersion parameters have been identified previously by mechanical experiments on passive cardiac tissue by Sommer et al. [94] and are set to *κ*_f_ = 0.08 and *κ*_s_ = 0.09.

**Fig. 13:**
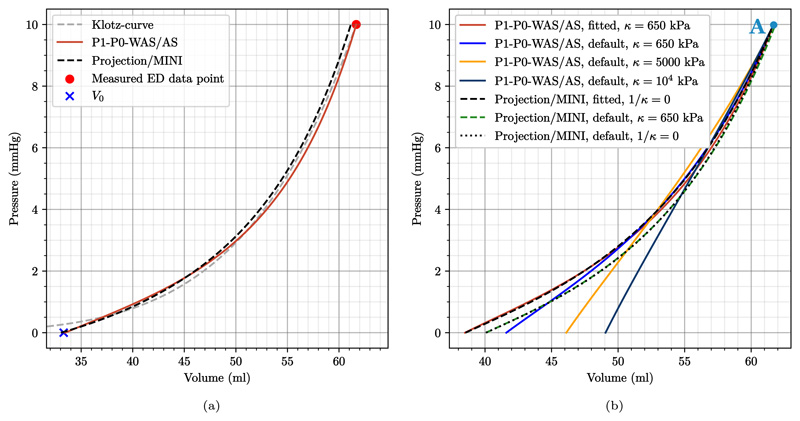
3D–0D model of the heart Passive model calibration and unloading following Marx et al. [108]. (a) An ex-vivo setup was used to calibrate the passive material parameters to fit the EDPVR of Klotz et al. [107]; (b) Calibrated as well as default material parameters, see Table 3, were used in an in-vivo setup to generate prestress using a backward-displacement scheme. Shown are the final loading curves. Note that loading curves for P1–P0-WAS and P1–P0-AS coincide and are thus only given once. The same applies for projection-stabilized and MINI elements. The marker “A” denotes the time-point for stress plots in Figs. 16 and 18.

To eliminate potential differences in stress/strain due to parameterization we fitted parameters to achieve similar pressure–volume (PV) loops and a similar end-diastolic PV relationship (EDPVR) for all element types. First, initial passive material parameters above were fitted to the empiric description of the EDPVR by Klotz et al. [107]. For each element type we used a backward displacement algorithm and boundary conditions replicating experiments in [107] according to Marx et al. [108], see Fig. 13. This fitting resulted in multiplicative scaling factors of 0.4529 (P1–P0 elements) and 0.9582 (locking-free elements) for the stress-like material parameters (*a*, *a*_f_, *a*_s_, *a*_fs_); and in multiplicative scaling factors of 1.0322 (P1–P0 elements) and 0.7981 (locking-free elements) for the dimensionless parameters (*b*, *b*_f_, *b*_s_, *b*_fs_). The list of fitted passive parameters is given in Table 3.

Active stress parameters were fitted using locking-free elements to reach a target peak pressure of 105mmHg in the LV. Using the same active stress parameters, the simulations with P1–P0 elements resulted in slightly higher peak pressure, 109.2 mmHg, see Fig. 15 (a). The end-diastolic state remained almost unchanged while the ejection fraction increased slightly. We attribute this to a higher contractility of the elements when the tissue is not modeled as a fully incompressible continuum. To achieve similar PV loops for P1–P0 elements, simulations were repeated with reduced active tension $T_{\text{ref}}^{•}.$ See Table 3 for a summary of all active stress parameters. Simulations parameters of the circulatory system were set as in Augustin et al. [71] with a cycle length of 0.585 s.

#### Results

First, simulation results in Fig. 13 (a) show that the passive parameterization – that was performed individually for all element types – allowed to reach the predicted stress-free volume and the given end-diastolic volume almost perfectly while reproducing the shape of the Klotz EDPVR curve. Boundary conditions for this experiment correspond to the *ex-vivo* setting described above replicating passive inflation experiments [107]. With fitted material parameters we repeat the backward displacement algorithm to get a stress-free reference geometry with *in-vivo* boundary conditions to model the constraints imposed by the pericardium. Loading this stress-free configuration to end-diastolic pressure results in loading curves as shown in Fig. 13 (b). When using fitted parameters, these loading curves are almost identical for all element types. Comparing to results obtained using default parameters from Gültekin et al. [97], we get quite similar loading curves using stabilized elements. This is expected as the values of the fitted parameters, see Table 3, are close to the default values obtained from triaxial shear tests [97]. Further, we can see that the choice of k has little influence on the loading for stabilized elements: using *κ* = 650 kPa or 1/*κ* = 0 results in coinciding curves. On the other hand, for P1–P0 elements the choice of *κ* has a clear impact: in line with expectations, higher values of *κ* result in a stiffer behavior of the tissue. Looking at stresses after the initial loading phase, marked by the letter “A” in Fig. 13 (b), we also see that for stabilized elements fitted and default parameters produce similar results and also the influence of the choice of *κ* is negligible, see Figs. 16 and 18. For P1–P0 elements, however, the stress fields are generally less smooth compared to stabilized elements and the dependency on κ is clearly visible.

**Fig. 14:**
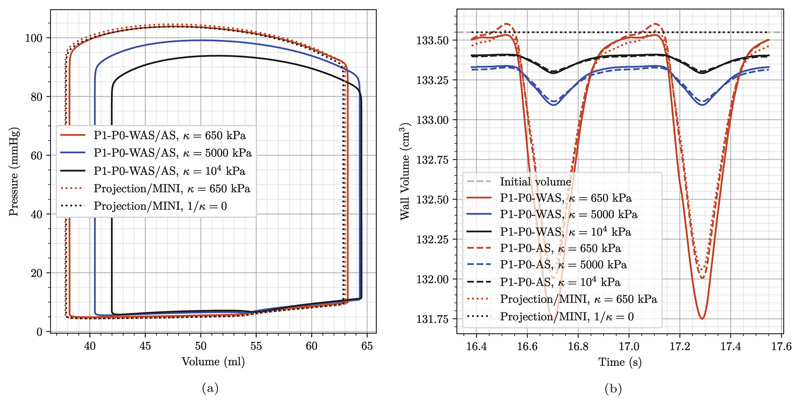
3D–0D model of the heart Comparison of approaches and influence of the bulk modulus *κ* using default material parameters from Table 3. (a) Converged left-ventricular PV loops. While the choice of *κ* has a high influence on the outcome when using P1–P0 elements it is negligible when using the proposed stabilized elements. Note that PV loops for P1–P0-WAS and P1–P0-AS coincide and are thus only given once. The same applies for projection-stabilized and MINI elements. (c) Change in tissue volume over the last two beats. As expected, higher values of *κ* lead to less myocardial volume change. The choice of 1/*κ* = 0 for projection-stabilized and MINI elements renders the material incompressible and the myocardial volume does not deviate from its initial value.

The pre-stressed configuration at marker “A” matches the geometry obtained from imaging and serves as the starting point for EM heart beat experiments as described in the following. To get to a converged solution of the closed-loop 3D–0D system we simulated 30 heart beats for each FE setting: 18 initialization beats with 1 Newton step corresponding to a semi-implicit (linearly-implicit) discretization method [109]; 10 beats with 2 Newton steps which is required to get a correct update due to the velocity dependence of the active-stress model; and two final beats with a fully converged Newton with a relative error reduction of the residual of *ϵ* = 10^−6^. First, using default passive parameters we again see a clear influence of the bulk modulus *κ* when using P1–P0 elements while for stabilized elements PV loops almost coincide for different values of *κ*, see Fig. 14 (a).

Looking at myocardial mass in Fig. 14 (b) it shows that for projection-stabilized and MINI elements the mass remains unchanged at the initial value of 133.56 cm^3^ all the time when the tissue is modeled to be fully incompressible (1/*κ* = 0). Using a smaller value of *κ* = 650 kPa the tissue behaves nearly incompressible and especially during the ejection phase the myocardial mass decreases slightly by around 1.2%. This is about the same volume change that we see for P1–P0-AS elements using a penalty formulation with the same *κ* = 650 kPa. Compared to this, P1–P0-WAS elements result in a minimally larger volume change of around 1.4%.

**Fig. 15:**
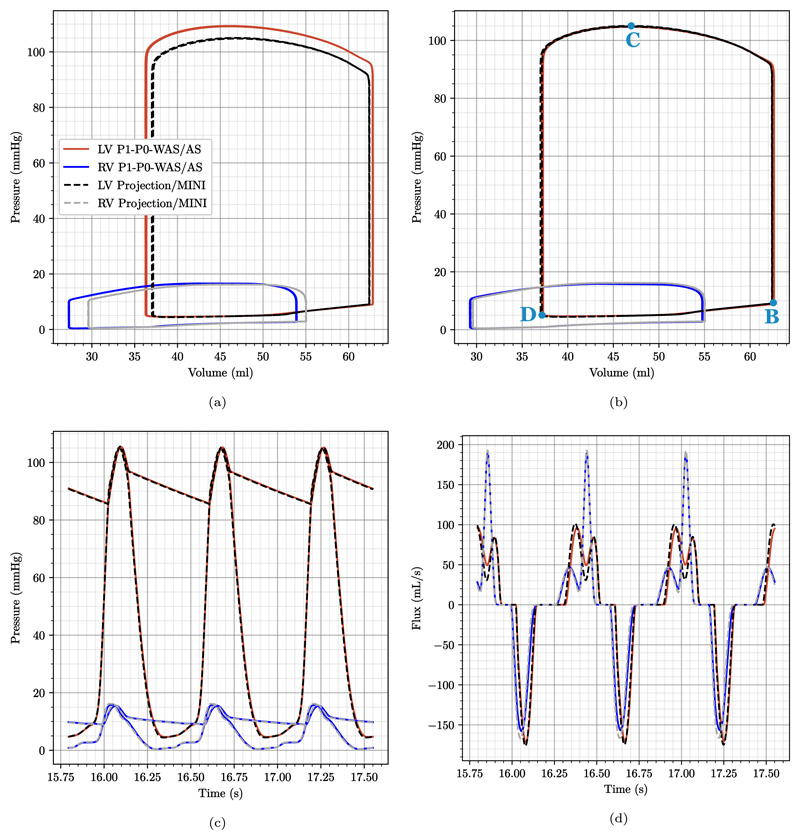
3D–0D model of the heart Plot comparing data traces for the P1–P0-WAS formulation (LV: red lines, RV: blue lines) and for the MINI and stabilized P1–P1 elements (LV: black dashed lines, RV: gray dashed lines). Shown are the last 3 beats of the simulation with (a) same active stress and preload parameters for P1–P0-WAS and locking free elements and (b–d) modified active stress and preload parameters for P1–P0-WAS elements to obtain matching PV loops. In particular we show: (a,b) converged PV-loops for both ventricles; (c) pressure trace for the LV and RV and pressure in the respective outflow vessel; (d) in- (negative values) and outflow (positive values) traces of both ventricles. “B”, “C”, “D” mark time-points for stress plots in Figs. 17 and 19.

**Fig. 16:**
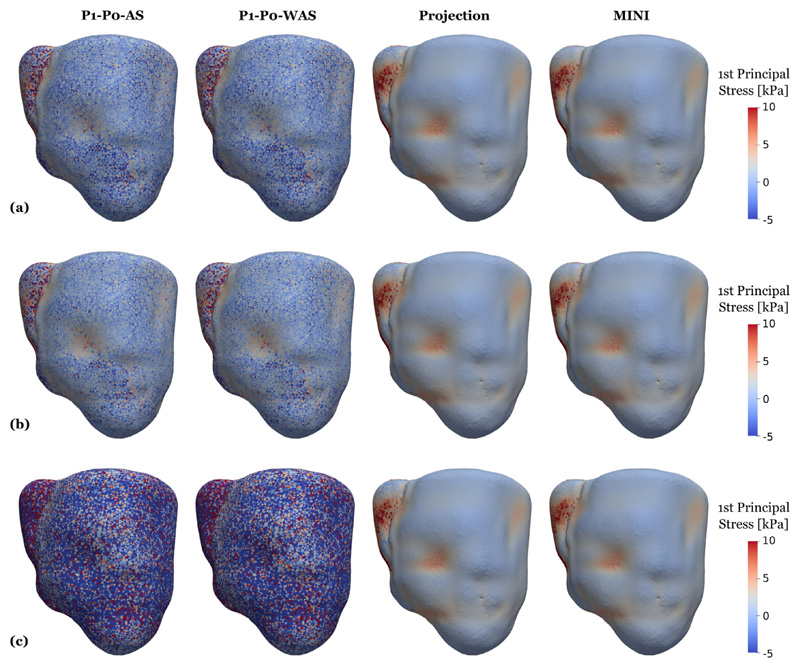
3D–0D model of the heart Snapshots of first principal stress values after loading, see Fig. 15 marker “A”. Compared are P1–P0-AS elements (first column), P1–P0-WAS elements (second column) stabilized P1–P1 elements (third column), and MINI elements (fourth column). Shown is element-wise passive stress which is not smoothed at the (a) loaded state using fitted parameters given in Table 3; (b) loaded state using default parameters given in Table 3 and a bulk modulus of *κ* = 650 kPa for all element types; (c) loaded state using default parameters given in Table 3 and a bulk modulus of *κ*= 5000 kPa for P1–P0 elements and 1/*κ* = 0 for projection-stabilized and MINI elements.

Further, see Fig. 15 (a), for a comparison of the final three PV loops using fitted passive but the same active stress parameters which resulted in higher pressures for P1–P0 elements. In Fig. 15 (b)-(d) we show traces for the refitted active stress parameters as described above. Here, the final three PV loops in Fig. 15 (b) coincide. Hence, the solution is converged and there is also no difference between the simulation with two Newton steps and the fully converged Newton method. This also holds true for pressures in the ventricles and adjacent arteries, see Fig. 15 (c), in- and outflow traces Fig. 15 (d), and strains/stresses.

**Fig. 17:**
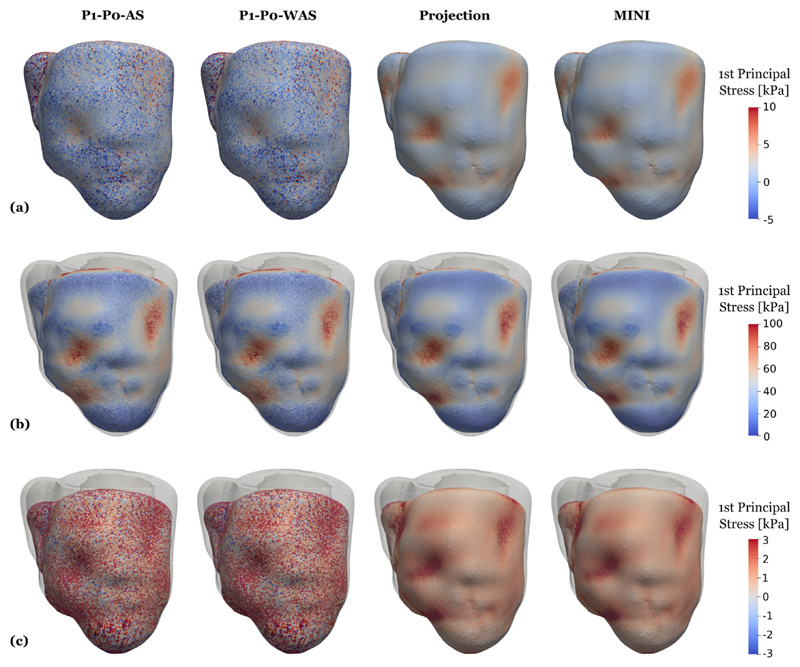
3D–0D model of the heart Snapshots of first principal stress values at end-diastole (first row), peak systole (second row), and at mitral valve opening (third row), see Fig. 15 “B”, “C”, “D” for a visualization of the time-points. Shown is element-wise total stress which is not smoothed. Gray outlines indicate the end-diastolic configuration. Compared are P1–P0-AS elements (first column), P1–P0-WAS elements (second column) stabilized P1–P1 elements (third column), and MINI elements (fourth column).

**Fig. 18:**
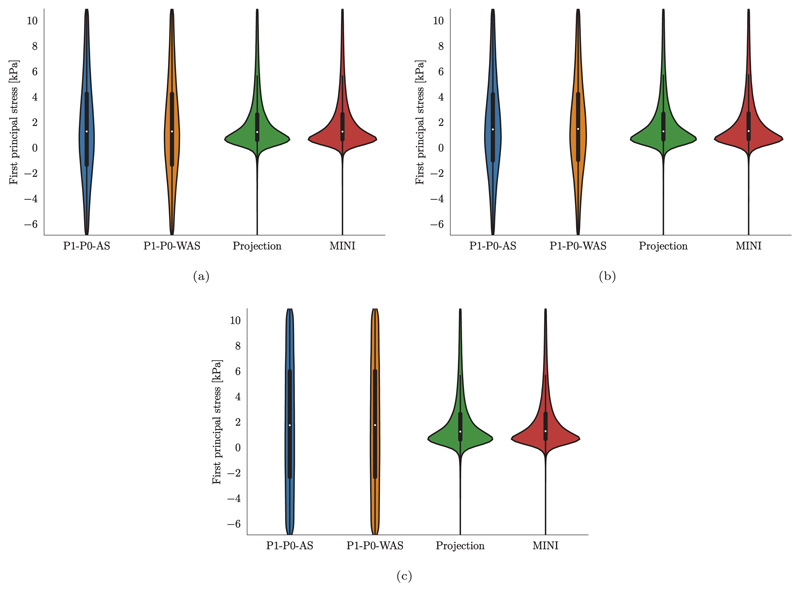
3D–0D model of the heart Violin plots of the first principal stress distribution after loading (a) loaded state using fitted parameters given in Table 3; (b) loaded state using default parameters given in Table 3 and a bulk modulus of *κ* = 650 kPa for all element types; (c) loaded state using default parameters given in Table 3 and a bulk modulus of *κ* = 5000 kPa for P1–P0 elements and 1/*κ* = 0 for projection-stabilized and MINI elements.

**Fig. 19:**
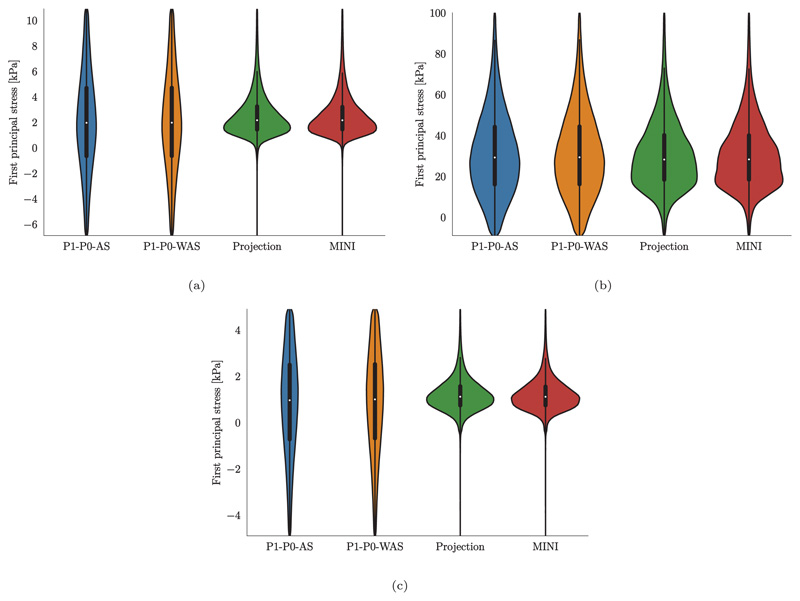
3D–0D model of the heart Violin plots of the first principal stress distribution at (a) end-diastole, (b) peak systole, (c) mitral valve opening.

In contrast to pressure, volume, and flow traces, stresses show a very different pattern when comparing locking-free to simple P1–P0 elements, see Fig. 17. In this plot we show element-wise, total first principal stress at three time points marked by B, C, and D in the PV loop in Fig. 15 (b) which represent B, the most expanded (end-diastole), C, the highest total stress (peak systole), and D, the most contracted (beginning of filling phase) states of the ventricles. Especially at end-diastole Fig. 17 (a) and the beginning of the filling phase Fig. 17 (c) where passive stress dominates over active stress we see a distinct checkerboard pattern for P1–P0 elements while solutions for projection-stabilized and MINI elements are smooth. Also in violin plots showing the stress distribution over the whole tissue domain we see a clear difference for these time points, see Fig. 19 (a) and (c). On the other hand, the stress distribution is very similar for all element types at peak-systole where active stress dominates, see Fig. 17 (b) and Fig. 19 (b).

#### Numerical performance

Computational times for the simulation using different element types are given in Table 4. Simulations were performed on 128 cores of Archer2 and we distinguish between solver-time, *t*_*s*,•_, the accumulated time of the linear solver (GMRES), and assembly-time, *t*_*a*,•_ the accumulated time of matrix and vector assembling of the linearized system (18)–(20).

In total, for a full simulation with loading, 18 initialization beats with 1 Newton step, 10 initialization beats with 2 Newton steps, and 2 final beats with a fully converging Newton method the computational costs were around 3.5 h for P1–P0 elements, 13 h for projection stabilized elements, and 17.5 h for MINI elements, see Table 4 for exact values. Here, in addition to GMRES solver and assembly times, also input–output times, the solution of the R–E model governing electrophysiology, ODE times, and postprocessing are taken into account. Using a coarser mesh with 45 686 elements and 11850 nodes tractable computational times could also be achieved on a desktop machine (AMD Ryzen Threadripper 2990X) with 2.5 min for one heart beat on 32 cores using P1–P0 elements and 7.15 minutes using locking-free projection stabilized elements. Total computational times on the desktop machine for 30 beats were 94.6minutes for P1–P0 and 264.4minutes for projection stabilized elements.

**Fig. 20:**
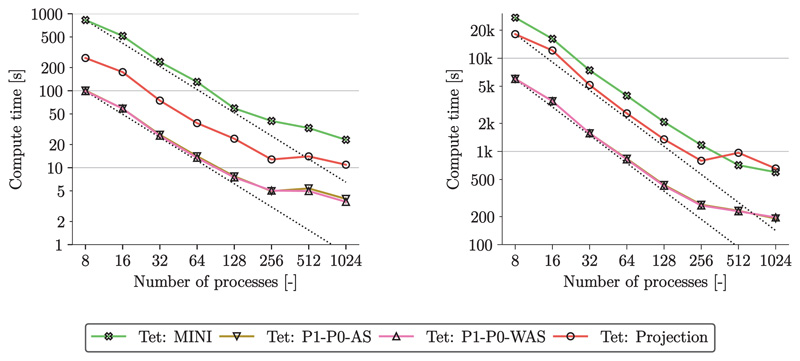
3D–0D model of the heart Strong scaling results for the loading phase (left) and one beat (right). Simulations were performed on 16 to 1024 cores on Archer2.

In Fig. 20 we show strong scaling properties of the simulation on 16 to 1024 cores of Archer2. Loading and heart beat experiments scale well up to 256 cores for all element types. For 512 and 1024 cores strong scaling efficiency drops markedly due to small local partition sizes (< 500 degrees of freedom per partition).

#### Discussion

In this benchmark, we show one of the most complete model of cardiac EM that is currently available: (i) Cardiac electrophysiology was modeled by a reaction-Eikonal model which predicts potential fields with high fidelity even on coarser grids [85]. (ii) Cellular dynamics were modeled by the physiological Grandi–Pasqualini–Bers model [98] which is coupled to the Land–Niederer model [99], representing human ventricular electrophysiology and active stress generation. This allows for strong coupling, i.e., to account for length and velocity effects on the cytosolic calcium transient, using an approach as described in Augustin et al. [83]. (iii) Passive tissue mechanics was modeled by the recent Holzapfel–Ogden type model [97] and active stress according to [101] using a recent approach by Regazzoni and Quarteroni [100] to avoid oscillations. Note that both, passive and active stress computation, account for fiber dispersion, hence, allowing to model the active tension generated by dispersed fibers. (iv) Spatially varying Robin boundary conditions were included to model the effect of the pericardium [105]. (v) The 3D PDE model was coupled to the physiologically comprehensive 0D closed-loop model CircAdapt of the cardiovascular system. This allows to replicate physiological behaviors under experimental standard protocols altering loading conditions and contractility [71]. (vi) Simulations were performed using locking-free finite elements – as presented in this paper – to accurately compute stress distributions.

All computational models of cardiac EM presented in the literature so far, e.g., [28,71,95,110–116], are missing one or mostly more of the above points. While the importance of model components (i)–(v) was discussed extensively in references above we could show in this paper that also (vi) is necessary to compute accurate stress fields in the tissue. Depending on the application this could be a very critical modeling component, e.g., for the accurate prediction of rupture risks or for the estimation of growth and remodeling based on stress. Note that also for this benchmark we see no substantial difference between simulation outcomes using standard P1–P0-AS and the P1–P0-WAS formulation; both approaches fail to match stress results from gold-standard elements.

While stress fields differ vastly the PV loops computed with locking-free and simple P1–P0 elements are very similar; at least if the bulk modulus *κ* is not too large, see Fig. 14 (a), or if active and passive tissue parameters are fitted independently for each element type, see Fig. 15 (b). For the fitting of passive parameters to the Klotz curve, which is state-of-the-art in recently published EM models [14,28,71] it shows that using P1–P0 elements correspond to a softer material. Here, the fitting compensates volumetric locking effects to a certain degree. On the other hand, the reference peak tension parameter $(T_{\text{ref}}^{•})$ had to be slightly reduced to reach the same target value as projection stabilized and MINI elements. As PV loops for default parameters coincide in Fig. 14 (a), we attribute the higher active stress generation in this case to the softer passive material and hence, a reduced resistance of the ground matrix material to compression in fiber direction. However, when using default parameters or fitted parameters for active and passive tissue properties, PV traces for the different element types are almost identical. This shows that simple P1–P0 elements can predict most simulation outputs as good as gold standard formulations and are thus adequate for cardiac EM simulations when stresses are not a primary quantity of interest. In this case, the computational efficiency of P1–P0 elements might trump the numerical accuracy of locking-free elements.

High resolution EM models require efficient numerical solvers to limit the computational cost that results from a high number of degrees of freedom to capture anatomical details as well as a high number of time steps. Strong scaling characteristics of our EM framework were reported in detail previously [83,117]. In the present work, we showed that strong scaling is preserved when using locking free elements and coupling to a 0D model of the circulatory system.

Using the advanced approach presented in this paper, the time needed for the passive filling of the bi-ventricular model of the heart is very low. For the projection stabilized element loading times are less than half a minute on 128 cores@2.25 GHz of Archer2 (Table 4) for the simulation with 444936 degrees of freedom. Even on 8 cores the passive filling could be achieved within 5 minutes (Fig. 20). In comparison, a recent work [21] reports compute times for a similar passive inflation scenario using locking-free elements that were around 162 min (203 214 degrees of freedom, 8 cores@2.8 GHz). Fast loading times are crucial for the estimation of the stress-free reference configuration using fixed-point iterations [118,119].

Computational cost for one heart beat – using grids with a comparable number of elements and nodes but, in general, P1–P0 elements – range from 1.8 to 24 h in previous studies [95,111,112,114,115]. In this work, we could show that even with locking-free elements one heart beat can be simulated within 27 min on 128 cores of Archer2, see Table 4, and within 11 min using 1024 cores, see Fig. 20. Still, P1–P0 elements are computationally less expensive with one heart beat in around 7 min using 128 cores and 3.2 min using 1024 cores. Using a coarser mesh as in [71] fast computational times are also possible on desktop machines with 2.5 min for one heart beat on 32 cores using P1–P0 elements and 7.15 min using locking-free elements. This computational efficiency is of paramount importance for future parameterization studies where numerous forward simulations have to be carried out to personalize models to patient data.

#### Limitations

First, we set an arbitrary number of 30 heart beats which was more than enough to reach a limit cycle in all experiments. However, an automatic stopping criterion could be used that stops the simulation after reaching the limit cycle. Simulation times could be further reduced by accelerating the convergence to a limit cycle using data-driven 0D emulators [120] or by tuning the 0D CircAdapt model to predict PV traces from the 3D-0D model more effectively, hence, reducing the number of beats needed to a converged solution.

Second, the parameterization of the model has room for improvement. Passive parameters were fitted to the empiric Klotz curve using end-diastolic volume and pressure and active stress was fitted using a target peak pressure value. Other model components such as the reaction-Eikonal model and CircAdapt were not parameterized to fit experimental data, default values from the literature were used. The personalization of the complete model to patient-specific data is not within the scope of this contribution, however, the computational efficiency of the model is of crucial importance for parameter identification studies that often require a large number of forward simulations.

Third, no independent validation of the model is performed. This could be done by comparing displacements or strains predicted by the model to observations from cine MRI or 3D tagged MRI data, see, e.g., [14,114,121]. However, in this work, we focused on showing advantages of locking-free elements for applied simulations using an advanced setup which is necessary to replicate physiological behavior. A rigorous, independent validation as described above will be the focus of future studies.

Finally, locking-free formulations as presented in this paper require the solution of a block system, which in turn necessitates suitable preconditioning for computational efficiency. This is not a trivial task, however, preconditioners used for simulations in this paper are publicly available through the open-source software framework *PETSc*.

## 4 Conclusion

In this study, we introduced stabilization techniques that accelerate simulations of elastic materials, in particular, we apply the methods to model nearly and fully incompressible fiber-reinforced solids such as arterial wall or myocardial tissue. A MINI element formulation and a simple and computationally efficient technique based on a local pressure projection were presented. Both methods were applied for the first time for simulations of anisotropic materials and showed to be an excellent choice when the use of higher order or Taylor–Hood elements is not desired. This is the case, e.g., for detailed, high-resolution problem domains that results in a high number of degrees of freedom. We showed that both approaches are very versatile and can be applied to stationary and transient problems as well as hexahedral and tetrahedral grids without modifications. It is worth noting that all required implementations are purely on the element level, thus, facilitating an inclusion in existing FE codes. Furthermore, solvers and preconditioners used to solve the linearized block system of equations are available through the open-source software package *PETSc* [87].

We showed the robustness and accuracy of the chosen approaches in two benchmark problems from the literature: first, a thick-walled cylindrical tube representing arterial tissue and second, an ellipsoid representing LV myocardial tissue. Additionally, in a third application of the stabilization approaches, we presented a complex 3D–0D model of the ventricles. This constitutes a highly advanced computational EM model of the heart where all components are captured by physiological, state-of-the-art models. We could show that for the first time accurate and physiologically detailed cardiovascular simulations are feasible within a clinically tractable time frame.

Computational efficiency of the methods is unprecedented in the literature and the framework shows excellent strong scaling on desktop and HPC architectures. The high versatility of the one-fits-all approach allows the simulation of nearly and fully incompressible fiber-reinforced materials in many different scenarios. Overall, this offers the possibility to perform accurate simulations of biological tissues in clinically tractable time frames, also enabling parameterization studies where numerous forward simulations have to be carried out to personalize models to patient data.

## Supplementary Material

Appendix

## Figures and Tables

**Table 1:** *Artery benchmark*: properties of idealized artery meshes used in Section 3.1.

𝓁	Elements (Hex)	Elements (Tet)	Nodes
1	960	5760	1320
2	7680	46080	9072
3	25920	155520	29016
4	61440	368640	66912
5	120000	720000	128520
6	207360	1244160	219600
7	329280	1975690	345912

**Table 2:** *Ellipsoid benchmark:* properties of idealized ventricle meshes used in Section 3.2.

*ℓ*	Elements	Nodes
1	20709	4732
2	201495	38896
3	1747845	312784

**Table 3:** Summary of electrical and mechanical material parameters.

*Passive stress parameters: default [97]*							
*a* =	0. 4 kPa,	*a*_f_ =	3.05 kPa,	*a*_s_ =	1.25 kPa,	*a*_fs_ =	0.15 kPa,
*b* =	6.55 [–],	*b*_f_ =	29.05 [–],	*b*_s_ =	36.65 [–],	*b*_fs_ =	6.28 [–].

*Passive stress parameters: fitted for P1–P0 elements*							
*a* =	0.1812 kPa,	*a*_f_ =	1.3813 kPa,	*a*_s_ =	0.5661 kPa,	*a*_fs_ =	0.0679 kPa,
*b* =	6.7609 [–],	*b*_f_ =	29.9854 [–],	*b*_s_ =	37.8301 [–],	*b*_fs_ =	6.4822 [–].

*Passive stress parameters: fitted for stabilized elements*							
*a* =	0.3833 kPa,	*a*_f_ =	2.9225 kPa,	*a*_s_ =	1.1978 kPa,	*a*_fs_ =	0.1437 kPa,
*b* =	5.2278 [–],	*b*_f_ =	23.1848 [–],	*b*_s_ =	29.2504 [–],	*b*_fs_ =	5.0121 [–].

*Active stress parameters*							
$T_{\text{ref}}^{\text{LV}}=$	200.0 mN mm^−2^,	$T_{\text{ref}}^{\text{RV}}=$	160.0 mN mm^−2^,	[Ca^2+^]_T50_ =	0.52 *μ*molL^−1^,	TRPN_50_ =	0.37 [–],
*n*_TRPN_ =	1.54 [–],	*k*_TRPN_ =	0.14 ms^−1^,	*n*_xb_ =	3.38 [–],	*k*_xb_ =	4.9 × 10^−3^ ms^−1^.

*Adapted active stress parameters for P1–P0 elements*							
$T_{\text{ref}}^{\text{LV}}=$	190.0 mN mm^−2^,	$T_{\text{ref}}^{\text{RV}}=$	130.0 mN mm^−2^.				

**Table 4:** Summary of computational times on 128 cores of Archer2 for the different finite element types. Given are degrees of freedom (DOF) for the finest resolution bi-ventricular geometry as well as solver, assembly, and total computational times for one beat using one Newton iteration (*t*_s,1_, t_a,1_) two Newton iterations (*t*_s,2_, *t*_a,2_) and a fully converged Newton solution (*t*_s,*c*_, *t*_a,*c*_). *T*_b,1_, *T*_b,2_, and *T*_b,c_ correspond to the total simulation time per heart beat for one Newton iteration, two Newton iteration, and fully converged Newton scenarios. Timings refer to a single heart beat lasting 0.585 s at a time step size of 1 ms. In addition, the times required for the loading phase, *T*_Id_, and the total simulation times including 0D solution, IO, and postprocessing are presented. Note that for P1–P0 elements the hydrostatic pressure *p* is statically condensed on the element level and thus not considered for counting DOFs.

Type	DOF	*t*_s,1_/*t*_a,1_	*t*_s,2_/*t*_a,2_	*t*_s,c_/*t*_a,c_	*T*_b,1_*T*_b,2_/*T*_b,c_	*T*_ld_	Total
[–]	[–]	[s]	[s]	[s]	[s]	[s]	[–]
P1–P0-AS	333702	93.7/93.4	183.6/186.8	938.4/739.0	221.3/438.6/1947.5	7.71	207.2
P1–P0-WAS	333702	92.1/92.9	181.2/185.8	922.4/739.7	218.7/434.4/1930.4	7.48	205.0
Projection	444936	573.6/119.1	1289.1/238.2	7479.6/921.3	727.1/1606.0/8694.3	23.8	777.0
MINI	444936	600.8/495.2	1242.1/990.4	6126.3/3078.8	1181.8/2328.3/9547.5	59.1	1060.5
