# Supplementary material for: An accurate, robust, and efficient finite element framework with applications to anisotropic, nearly and fully incompressible elasticity
Source: Comput Methods Appl Mech Eng. Author manuscript; Available in PMC 2022 May 1. (PMC7612621; doi:10.1016/j.cma.2022.114887)
Supplement: Appendix [file EMS144165-supplement-Appendix.pdf]

Appendix A. Linearization

We will give a short summary of the linearization of a cavity volume V_{CAV} defined by

$$V_{\mathrm{CAV}} := \frac{1}{3}\int_{\Gamma_{\mathrm{CAV}}} \boldsymbol{x}\cdot\boldsymbol{n}\,\mathrm{d}s_{\boldsymbol{x}}.$$

Using Nanson's formula and $\boldsymbol{x} = \boldsymbol{X} + \boldsymbol{u}$ we can rewrite this as

$$V_{\mathrm{CAV}} = \frac{1}{3}\int_{\Gamma_{\mathrm{CAV},0}} (\boldsymbol{X}+\boldsymbol{u})\cdot J\boldsymbol{F}^{-\top}\boldsymbol{N}\,\mathrm{d}s_{\boldsymbol{X}}$$

Using the known linearizations

$$\frac{\partial J}{\partial \boldsymbol{F}} : \mathrm{Grad}\,\Delta\boldsymbol{u} = J\boldsymbol{F}^{-\top} : \mathrm{Grad}\,\Delta\boldsymbol{u} \tag{A.1}$$

$$\frac{\partial \boldsymbol{F}^{-\top}}{\partial \boldsymbol{F}} : \mathrm{Grad}\,\Delta\boldsymbol{u} = -\boldsymbol{F}^{-\top}(\mathrm{Grad}\,\Delta\boldsymbol{u})^{\top}\boldsymbol{F}^{-\top} \tag{A.2}$$

we can calculate the linearization around $\Delta\boldsymbol{u}$ as

$$d_{\mathrm{CAV}}(\boldsymbol{u};\,\Delta\boldsymbol{u}) := D_{\Delta\boldsymbol{u}}V_{\mathrm{CAV}} = D_{\Delta\boldsymbol{u}}\frac{1}{3}\int_{\Gamma_{\mathrm{CAV}}} \boldsymbol{x}\cdot\boldsymbol{n}\,\mathrm{d}s_{\boldsymbol{x}} \tag{A.3}$$

$$= D_{\Delta\boldsymbol{u}}\frac{1}{3}\int_{\Gamma_{\mathrm{CAV},0}} J\,(\boldsymbol{X}+\boldsymbol{u})\cdot\boldsymbol{F}^{-\top}\boldsymbol{N}\,\mathrm{d}s_{\boldsymbol{X}} \tag{A.4}$$

$$= \frac{1}{3}\int_{\Gamma_{\mathrm{CAV},0}} J(\boldsymbol{F}^{-\top} : \mathrm{Grad}\,\Delta\boldsymbol{u})\boldsymbol{x}\cdot\boldsymbol{F}^{-\top}\boldsymbol{N}\,\mathrm{d}s_{\boldsymbol{X}} \tag{A.5}$$

$$- \frac{1}{3}\int_{\Gamma_{\mathrm{CAV},0}} J\boldsymbol{x}\cdot\boldsymbol{F}^{-\top}(\mathrm{Grad}\,\Delta\boldsymbol{u})^{\top}\boldsymbol{F}^{-\top}\boldsymbol{N}\,\mathrm{d}s_{\boldsymbol{X}} \tag{A.6}$$

$$+ \frac{1}{3}\int_{\Gamma_{\mathrm{CAV},0}} J\Delta\boldsymbol{u}\cdot\boldsymbol{F}^{-\top}\boldsymbol{N}\,\mathrm{d}s_{\boldsymbol{X}} \tag{A.7}$$

Appendix B. Static condensation for inhomogeneous Neumann boundary condition

While homogeneous Neumann boundary conditions do not alter the process of static condensation, the procedure needs to be adapted to for inhomogeneous ones. First, looking at the definition of the nonlinear residual R_{vol} in (6) we see that this can be split as

$$R_{\mathrm{vol}} = R_{\mathrm{vol},\Omega_0} + R_{\mathrm{vol},\Gamma_{N,0}}$$

where $R_{\mathrm{vol},\Omega_0}$ holds all the terms coming from integration over the domain Ω_0 and $R_{\mathrm{vol},\Gamma_{N,0}}$ holds all the terms coming from integration over the Neumann surfaces. Next, note that the bubble functions $\hat{\psi}_{\mathrm{B}}$ for tetrahedral elements as well as their hexahedral counterparts $\hat{\psi}_{\mathrm{B},1}$, $\hat{\psi}_{\mathrm{B},2}$ have compact support in the FE interior. However, the respective gradients do not vanish on the FE boundary. Consider an arbitrary FE $K \in \mathcal{T}_h$ with $K \cap \Gamma_{N,0} \neq \emptyset$. The gradient of a bubble function occurs in bilinear-form a_{N}^k in (17), for the argument $\Delta \boldsymbol{u} \in V_h$. This yields a non-zero contribution to the element-stiffness-matrix, whereas there is no contribution from $R_{\mathrm{vol},\Gamma_{N,0}}$ to the total element residual vector. Using the decomposition of local degrees of freedom into *exterior*, E and *interior*, I it follows that the local block system can be written in the following form

$$\begin{pmatrix} \boldsymbol{K}_{\mathrm{EE}} + \boldsymbol{K}_{\Gamma,\mathrm{EE}} & \boldsymbol{K}_{\mathrm{EI}} + \boldsymbol{K}_{\Gamma,\mathrm{EI}} & \boldsymbol{B}_{\mathrm{E}} \\ \boldsymbol{K}_{\mathrm{IE}} & \boldsymbol{K}_{\mathrm{II}} & \boldsymbol{B}_{\mathrm{I}} \\ \boldsymbol{C}_{\mathrm{E}} & \boldsymbol{C}_{\mathrm{I}} & \boldsymbol{D}_{\mathrm{E}} \end{pmatrix} \begin{pmatrix} \Delta \underline{u}_{\mathrm{E}} \\ \Delta \underline{u}_{\mathrm{I}} \\ \Delta \underline{p}_{\mathrm{E}} \end{pmatrix} = \begin{pmatrix} -\underline{R}_{\mathrm{vol,E}} - R_{\mathrm{vol},\Gamma,\mathrm{E}} \\ -\underline{R}_{\mathrm{vol,I}} \\ -\underline{R}_{\mathrm{inc,E}} \end{pmatrix}.$$

The interior degrees of freedom can be statically condensed. On element level this leads to the static condensed system

$$\underbrace{\begin{pmatrix} \widetilde{\boldsymbol{K}} & \widetilde{\boldsymbol{B}} \\ \widetilde{\boldsymbol{C}} & \widetilde{\boldsymbol{D}} \end{pmatrix}}_{:=\widetilde{\boldsymbol{A}}} \begin{pmatrix} \Delta \underline{u}_{\mathrm{E}} \\ \Delta \underline{p}_{\mathrm{E}} \end{pmatrix} + \underbrace{\begin{pmatrix} \widetilde{\boldsymbol{K}}_{\Gamma} & \widetilde{\boldsymbol{B}}_{\Gamma} \\ \boldsymbol{0} & \boldsymbol{0} \end{pmatrix}}_{:=\widetilde{\boldsymbol{A}}_{\Gamma}} \begin{pmatrix} \Delta \underline{u}_{\mathrm{E}} \\ \Delta \underline{p}_{\mathrm{E}} \end{pmatrix} = \underbrace{\begin{pmatrix} -\widetilde{\underline{R}}_{\mathrm{vol}} \\ -\widetilde{\underline{R}}_{\mathrm{inc}} \end{pmatrix}}_{:=-\underline{R}} + \underbrace{\begin{pmatrix} -\widetilde{\underline{R}}_{\Gamma,\mathrm{upper}} \\ \underline{0} \end{pmatrix}}_{:=-\underline{R}_{\Gamma}},$$

where

$$\begin{aligned} \widetilde{\boldsymbol{K}} &:= \boldsymbol{K}_{\mathrm{EE}} - \boldsymbol{K}_{\mathrm{EI}} \boldsymbol{K}_{\mathrm{II}}^{-1} \boldsymbol{K}_{\mathrm{IE}}, & \widetilde{\boldsymbol{B}} &:= \boldsymbol{B}_{\mathrm{E}} - \boldsymbol{K}_{\mathrm{EI}} \boldsymbol{K}_{\mathrm{II}}^{-1} \boldsymbol{B}_{\mathrm{I}}, \\ \widetilde{\boldsymbol{C}} &:= \boldsymbol{C}_{\mathrm{E}} - \boldsymbol{C}_{\mathrm{I}} \boldsymbol{K}_{\mathrm{II}}^{-1} \boldsymbol{K}_{\mathrm{IE}}, & \widetilde{\boldsymbol{D}} &:= \boldsymbol{D}_{\mathrm{E}} - \boldsymbol{C}_{\mathrm{I}} \boldsymbol{K}_{\mathrm{II}}^{-1} \boldsymbol{B}_{\mathrm{I}}, \\ \widetilde{\boldsymbol{K}}_{\Gamma} &:= \boldsymbol{K}_{\Gamma,\mathrm{EE}} - \boldsymbol{K}_{\Gamma,\mathrm{EI}} \boldsymbol{K}_{\mathrm{II}}^{-1} \boldsymbol{K}_{\mathrm{IE}}, & \widetilde{\boldsymbol{B}}_{\Gamma} &:= -\boldsymbol{K}_{\Gamma,\mathrm{EI}} \boldsymbol{K}_{\mathrm{II}}^{-1} \boldsymbol{B}_{\mathrm{I}}, \\ \widetilde{\underline{R}}_{\mathrm{vol}} &:= \underline{R}_{\mathrm{vol,E}} - \boldsymbol{K}_{\mathrm{EI}} \boldsymbol{K}_{\mathrm{II}}^{-1} \underline{R}_{\mathrm{vol,I}}, & \widetilde{\underline{R}}_{\Gamma,\mathrm{vol}} &:= \underline{R}_{\Gamma,\mathrm{E}} - \boldsymbol{K}_{\Gamma,\mathrm{EI}} \boldsymbol{K}_{\mathrm{II}}^{-1} \underline{R}_{\mathrm{vol,I}}, \\ \widetilde{\underline{R}}_{\mathrm{inc}} &:= \underline{R}_{\mathrm{inc,E}} - \boldsymbol{C}_{\mathrm{I}} \boldsymbol{K}_{\mathrm{II}}^{-1} \underline{R}_{\mathrm{inc,I}}. \end{aligned}$$

The individual element matrices/vectors $\widetilde{\boldsymbol{A}}$, $\widetilde{\boldsymbol{A}}_{\Gamma}$, $\widetilde{\underline{R}}$, and $\widetilde{\underline{R}}_{\Gamma}$ can be assembled into a global stiffness matrix through loops over volume elements and surface elements respectively. In the case of an attached circulatory system a static condensation can be performed in an analogous way

$$\begin{pmatrix} \boldsymbol{K}_{\mathrm{EE}} + \boldsymbol{K}_{\Gamma,\mathrm{EE}} & \boldsymbol{K}_{\mathrm{EI}} + \boldsymbol{K}_{\Gamma,\mathrm{EI}} & \boldsymbol{B}_{\mathrm{E}} & \boldsymbol{E}_{\mathrm{CAV,E}} \\ \boldsymbol{K}_{\mathrm{IE}} & \boldsymbol{K}_{\mathrm{II}} & \boldsymbol{B}_{\mathrm{I}} & \boldsymbol{0} \\ \boldsymbol{C}_{\mathrm{E}} & \boldsymbol{C}_{\mathrm{I}} & \boldsymbol{D}_{\mathrm{E}} & \boldsymbol{0} \\ \boldsymbol{F}_{\mathrm{CAV,E}} & \boldsymbol{F}_{\mathrm{CAV,I}} & \boldsymbol{0} & \boldsymbol{G}_{\mathrm{CAV}} \end{pmatrix} \begin{pmatrix} \Delta \underline{u}_{\mathrm{E}} \\ \Delta \underline{u}_{\mathrm{I}} \\ \Delta \underline{p}_{\mathrm{E}} \\ \Delta \underline{p}_{\mathrm{CAV}} \end{pmatrix} = \begin{pmatrix} -\underline{R}_{\mathrm{vol,E}} - R_{\Gamma,\mathrm{vol,E}} \\ -\underline{R}_{\mathrm{vol,I}} \\ -\underline{R}_{\mathrm{inc,E}} \\ -\underline{R}_{\mathrm{CAV,E}} \end{pmatrix},$$

where we assumed $n_{\mathrm{CAV}} = 1$ for brevity of presentation. The generalization to $n_{\mathrm{CAV}} > 1$ is straightforward. Static condensation of all interior degrees of freedom leads to

$$\underbrace{\begin{pmatrix} \widetilde{\boldsymbol{K}} & \widetilde{\boldsymbol{B}} & \boldsymbol{E}_{\mathrm{CAV,E}} \\ \widetilde{\boldsymbol{C}} & \widetilde{\boldsymbol{D}} & \boldsymbol{0} \\ \widetilde{\boldsymbol{F}}_{\mathrm{CAV}} & \widetilde{\boldsymbol{H}}_{\mathrm{CAV}} & \boldsymbol{G}_{\mathrm{CAV}} \end{pmatrix}}_{:=\widetilde{\boldsymbol{A}}} \begin{pmatrix} \Delta \underline{u}_{\mathrm{E}} \\ \Delta \underline{p}_{\mathrm{E}} \\ \Delta \underline{p}_{\mathrm{CAV}} \end{pmatrix} + \underbrace{\begin{pmatrix} \widetilde{\boldsymbol{K}}_{\Gamma} & \widetilde{\boldsymbol{B}}_{\Gamma} & \boldsymbol{0} \\ \boldsymbol{0} & \boldsymbol{0} & \boldsymbol{0} \\ \boldsymbol{0} & \boldsymbol{0} & \boldsymbol{0} \end{pmatrix}}_{:=\widetilde{\boldsymbol{A}}_{\Gamma}} \begin{pmatrix} \Delta \underline{u}_{\mathrm{E}} \\ \Delta \underline{p}_{\mathrm{E}} \\ \Delta \underline{p}_{\mathrm{CAV}} \end{pmatrix} = \underbrace{\begin{pmatrix} -\widetilde{\underline{R}}_{\mathrm{vol}} \\ -\widetilde{\underline{R}}_{\mathrm{inc}} \\ \widetilde{\underline{R}}_{\mathrm{CAV}} \end{pmatrix}}_{:=-\underline{R}} + \underbrace{\begin{pmatrix} -\widetilde{\underline{R}}_{\Gamma,\mathrm{vol}} \\ \underline{0} \\ \underline{0} \end{pmatrix}}_{:=-\underline{R}_{\Gamma}},$$

where

$$\begin{aligned} \widetilde{\boldsymbol{F}}_{\mathrm{CAV}} &:= \boldsymbol{F}_{\mathrm{CAV,E}} - \boldsymbol{F}_{\mathrm{CAV,I}} \boldsymbol{K}_{\mathrm{II}}^{-1} \boldsymbol{K}_{\mathrm{IE}}, \\ \widetilde{\boldsymbol{H}}_{\mathrm{CAV}} &:= -\boldsymbol{F}_{\mathrm{CAV,I}} \boldsymbol{K}_{\mathrm{II}}^{-1} \boldsymbol{B}_{\mathrm{I}}, \\ \widetilde{\underline{R}}_{\mathrm{CAV}} &:= \underline{R}_{\mathrm{CAV}} - \boldsymbol{F}_{\mathrm{CAV,I}} \boldsymbol{K}_{\mathrm{II}}^{-1} \underline{R}_{\mathrm{vol,I}} \end{aligned}$$

Appendix C. Tensor calculus

We use the following results from tensor calculus, for more details we refer to, e.g., [68,122].

$$\frac{\partial \overline{\boldsymbol{C}}}{\partial \boldsymbol{C}} = J^{-\frac{2}{3}} \mathbb{P} \quad \text{with the projection tensor } \mathbb{P} := \mathbb{I} - \frac{1}{3} \boldsymbol{C}^{-1} \otimes \boldsymbol{C},$$

$$\frac{\partial \boldsymbol{C}^{-1}}{\partial \boldsymbol{C}} = -\boldsymbol{C}^{-1} \odot \boldsymbol{C}^{-1},$$
$$(\boldsymbol{A} \odot \boldsymbol{A})_{ijkl} := \frac{1}{2}\left(A_{ik}A_{jl} + A_{il}A_{jk}\right).$$

For symmetric $\boldsymbol{A}$ it holds

$$\mathbb{P} : \boldsymbol{A} = \mathrm{Dev}(\boldsymbol{A}) = \boldsymbol{A} - \frac{1}{3}(\boldsymbol{A} : \boldsymbol{C})\boldsymbol{C}^{-1}$$

with the deviatoric operator in the Lagrangian description

$$\mathrm{Dev}(\bullet) = (\bullet) - \frac{1}{3}\left((\bullet) : \boldsymbol{C}\right)\boldsymbol{C}^{-1}. \tag{C.1}$$

The isochoric part of the second Piola–Kirchhoff stress tensor as well as the isochoric part of the fourth order elasticity tensor are given as

$$\boldsymbol{S}_{\mathrm{isc}} := 2\frac{\partial \overline{\Psi}(\overline{\boldsymbol{C}})}{\partial \boldsymbol{C}} = J^{-\frac{2}{3}}\mathrm{Dev}(\overline{\boldsymbol{S}}), \tag{C.2}$$
$$\overline{\boldsymbol{S}} := 2\frac{\partial \overline{\Psi}(\overline{\boldsymbol{C}})}{\partial \overline{\boldsymbol{C}}},$$
$$\mathbb{C}_{\mathrm{isc}} := 4\frac{\overline{\Psi}(\overline{\boldsymbol{C}})}{\partial \boldsymbol{C}\partial \boldsymbol{C}} = J^{-\frac{4}{3}}\mathbb{P}\overline{\mathbb{C}}\mathbb{P}^{\top} + J^{-\frac{2}{3}}\frac{2}{3}\mathrm{tr}(\boldsymbol{C}\overline{\boldsymbol{S}})\widetilde{\mathbb{P}} - \frac{4}{3}\boldsymbol{S}_{\mathrm{isc}} \overset{\mathrm{S}}{\otimes} \boldsymbol{C}^{-1}, \tag{C.3}$$
$$\overline{\mathbb{C}} := 4\frac{\partial \overline{\Psi}(\overline{\boldsymbol{C}})}{\partial \overline{\boldsymbol{C}}\partial \overline{\boldsymbol{C}}},$$
$$\widetilde{\mathbb{P}} := \boldsymbol{C}^{-1} \odot \boldsymbol{C}^{-1} - \frac{1}{3}\boldsymbol{C}^{-1} \otimes \boldsymbol{C}^{-1},$$
$$\boldsymbol{A} \overset{\mathrm{S}}{\otimes} \boldsymbol{B} := \frac{1}{2}\left(\boldsymbol{A} \otimes \boldsymbol{B} + \boldsymbol{B} \otimes \boldsymbol{A}\right).$$
